# Stabilization of Reversed Replication Forks by Telomerase Drives Telomere Catastrophe

**DOI:** 10.1016/j.cell.2017.11.047

**Published:** 2018-01-25

**Authors:** Pol Margalef, Panagiotis Kotsantis, Valerie Borel, Roberto Bellelli, Stephanie Panier, Simon J. Boulton

**Affiliations:** 1 The Francis Crick Institute, 1 Midland Road, London NW1 1AT, UK

**Keywords:** telomeres, telomerase, genome stability, Hoyeraal-Hreidarsson syndrome, replication fork reversal, RTEL1, PARP1, RECQ1, ZRANB3

## Abstract

Telomere maintenance critically depends on the distinct activities of telomerase, which adds telomeric repeats to solve the end replication problem, and RTEL1, which dismantles DNA secondary structures at telomeres to facilitate replisome progression. Here, we establish that reversed replication forks are a pathological substrate for telomerase and the source of telomere catastrophe in *Rtel1*^*−/−*^ cells. Inhibiting telomerase recruitment to telomeres, but not its activity, or blocking replication fork reversal through PARP1 inhibition or depleting UBC13 or ZRANB3 prevents the rapid accumulation of dysfunctional telomeres in RTEL1-deficient cells. In this context, we establish that telomerase binding to reversed replication forks inhibits telomere replication, which can be mimicked by preventing replication fork restart through depletion of RECQ1 or PARG. Our results lead us to propose that telomerase inappropriately binds to and inhibits restart of reversed replication forks within telomeres, which compromises replication and leads to critically short telomeres.

## Highlights

• Blocking telomerase recruitment rescues telomere catastrophe in *Rtel1*^*−/−*^ cells
• Reversed replication forks within telomeres are aberrantly bound by telomerase
• Blocking fork reversal rescues telomere catastrophe induced by telomerase
• Telomerase prevents restart of reversed replication forks at telomeres

## Graphical Abstract

**Figure:**
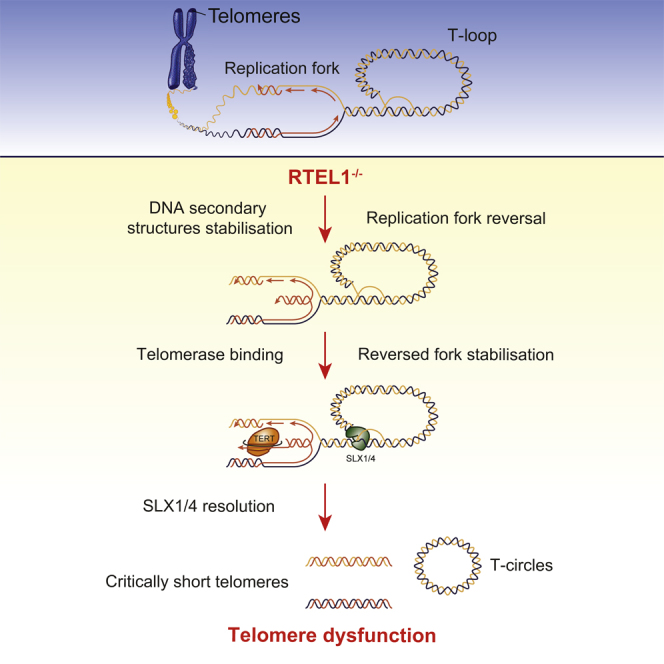

## Teaser

Telomerase can, paradoxically, contribute to telomere shortening by stabilizing stalled replication forks at chromosome ends.

## Introduction

Eukaryotic genomes are organized into linear chromosomes, which require the presence of telomeres to protect and maintain their chromosome ends. Telomeres are repetitive sequences (TTAGGG in vertebrates) that form complex nucleoprotein structures that protect chromosome ends from promiscuous DNA repair activities and nucleolytic degradation (reviewed in de Lange, 2005). Telomeres also shorten with each cell division due to processing by nucleases, genotoxic stress and the “end-replication problem” (Harley et al., 1990). To counteract telomere shortening, telomeric repeats can be extended by means of telomerase, a reverse transcriptase that uses an RNA moiety (TERC) as a template to extend the 3′ end of the chromosome (Greider and Blackburn, 1985, Shippen-Lentz and Blackburn, 1990). Telomerase expression is normally high in stem cells and repressed in somatic tissues but is frequently re-expressed in cancer cells as a means of maintaining telomere length during disease progression (Damle et al., 2004, Hultdin et al., 2003).

Telomere integrity is also dependent on the six-subunit Shelterin complex, which (1) suppresses ATM and ATR checkpoint signaling, (2) inhibits DNA double-strand break (DSB) repair pathways, and (3) facilitates semi-conservative telomere replication (de Lange, 2005). Shelterin is composed of telomere repeat-binding factor 1 and 2 (TRF1 and TRF2, respectively), protection of telomeres 1 (POT1), TRF1-interacting nuclear factor 2 (TIN2), repressor activator protein 1 (RAP1), and TPP1 (reviewed in Palm and de Lange, 2008). TRF1 is essential for efficient telomere replication, which averts telomeric fragility (Sfeir et al., 2009), whereas TRF2 prevents ATM activation at telomeres, telomere end-to-end fusions, and loss of the 3′ single-stranded overhang (Celli and de Lange, 2005, Karlseder et al., 1999, Sfeir and de Lange, 2012, van Steensel et al., 1998). Moreover, POT1 represses the activity of ATR at telomeres by binding to single-stranded DNA and thus preventing the recruitment of RPA (Denchi and de Lange, 2007, Takai et al., 2011).

One way that TRF2 protects chromosome ends is by promoting the formation of t-loops at telomeres, which form when the 3′ single-stranded telomeric G-rich overhang invades into internal telomeric repeats to form a lariat-like structure (Doksani et al., 2013, Griffith et al., 1999). By masking the processed DSB end within an internally protected structure, t-loops protect chromosome ends from degradation and deleterious DNA damage response (DDR) pathways (reviewed in O’Sullivan and Karlseder, 2010). Telomere replication and telomerase access to the 3′ telomeric end require that t-loops are transiently dismantled during S-phase, which is catalyzed by Regulator of Telomere Length 1 (RTEL1) (Sarek et al., 2015). In the absence of RTEL1, persistent t-loops are aberrantly excised by the SLX1/4 nuclease complex, leading to dramatic telomere length changes and loss of the t-loop as a circle (Vannier et al., 2012). Loss of RTEL1 also causes the appearance of telomeric fragility, which reflects a defect in unwinding telomeric G4-DNA structures that hinder DNA replication (Vannier et al., 2012). This second function of RTEL1 is also important genome-wide to facilitate processive genome replication and to prevent tumorigenesis (Vannier et al., 2013). The clinical importance of RTEL1’s role at telomeres was recently highlighted with the discovery that it is frequently mutated in Hoyeraal-Hreidarsson syndrome (HHS), a severe form of Dyskeratosis congenita (DKC) (Ballew et al., 2013a, Ballew et al., 2013b, Deng et al., 2013, Faure et al., 2014, Walne et al., 2013).

Paradoxically, the rapid onset of telomeric dysfunction following conditional murine RTEL1 inactivation does not result in chromosome end-to-end fusions nor activation of a robust DDR (Vannier et al., 2012). Since telomerase is constitutively active in mouse cells, it seemed plausible that this activity could act to heal critically short telomeres that arise in the absence of RTEL1, thereby preventing telomere fusions and induction of the DDR. However, we report here that eliminating telomerase activity does not lead to telomere fusions following inactivation of RTEL1. Strikingly, *Rtel1*^*−/−*^*Terc*^*−/−*^ cells are instead rescued for the rapid accumulation of dysfunctional telomeres normally observed following conditional loss of RTEL1, which implied that telomerase is driving telomere catastrophe in this context. We proceed to show that telomerase aberrantly accumulates at telomeres in the absence of RTEL1 and eliminating telomerase or blocking its recruitment to telomeres is sufficient to rescue telomere dysfunction in *Rtel1*-null cells. We present evidence that the abnormal association of telomerase with telomeres in these cells corresponds to its binding to single-ended DSBs generated at reversed replication forks that form as a consequence of persistent t-loops or unresolved telomeric G4-DNA structures. Consistent with this conclusion, blocking fork reversal is sufficient to rescue telomere dysfunction in *Rtel1*^*−/−*^ cells, whereas inhibiting the restart of reversed replication forks mimics the toxic effects of telomerase. These data reveal an unappreciated source of critically short telomeres that results from the aberrant binding and stabilization of reversed replication forks by telomerase.

## Results

### *Terc* Deletion Rescues Telomere Dysfunction in *Rtel1*-Deficient Cells

**Figure 1:**
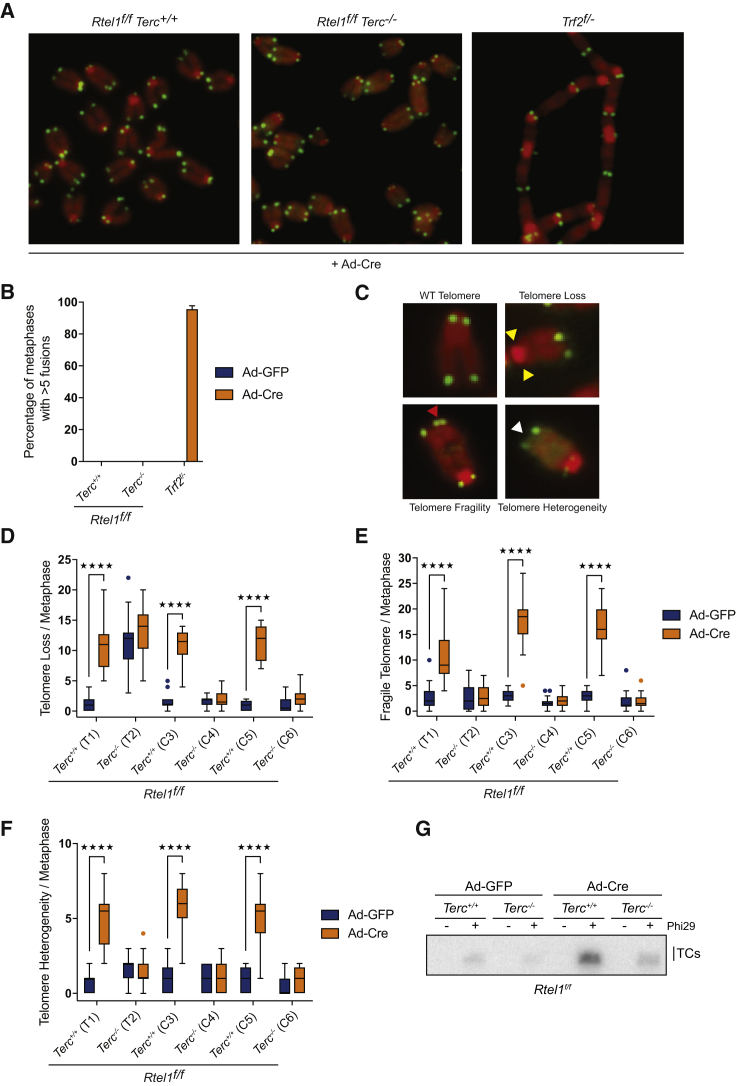
*Terc* Deletion Rescues Telomere Dysfunction in *Rtel1*-Deficient Cells (A) Telomeric FISH to analyze telomere fusions per metaphase in cells of the indicated genotypes 96 hr after Ad-Cre infection. Representative images of chromosomes from the different genotypes are shown. (B) Quantification of metaphases with more than 5 telomere fusions in cells of the indicated genotypes. Error bars, ±SD from three independent experiments. (C) Representative images of a wild-type chromosome and abnormal chromosomes with telomere loss (yellow arrows), telomere fragility (red arrows), and telomeric length heterogeneity (white arrows). (D–F) Quantification of telomere loss (D), telomere fragility (E), and telomere length heterogeneity (F) per metaphase 96 hr after Ad-GFP or Ad-Cre infection. Representative images of telomere FISH on metaphases are shown in Figure S1C. Boxplots represent the quantification from at least 30 metaphases from a representative experiment (^∗∗∗∗^p < 0.0001; two-way ANOVA). (G) Phi29-dependent telomere circles (TCs) from the indicated genotypes 96 hr after Ad-GFP or Ad-Cre infection. See also Figure S1.

**Figure S1:**
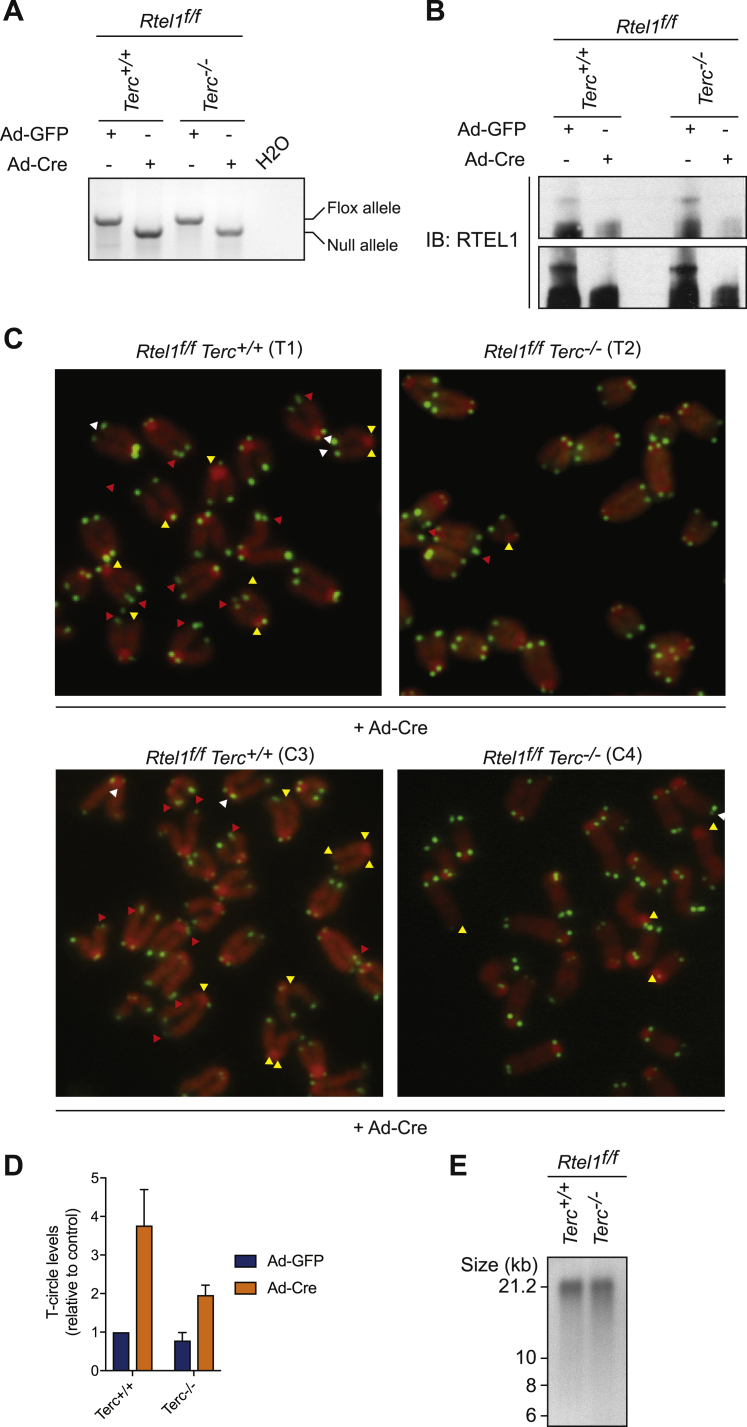
*Terc* Deletion Rescues Telomere Dysfunction in *Rtel1*-Deficient Cells, Related to Figure 1 (A) RTEL1 genotyping PCR on DNA derived from MAFs of the indicated genotypes 96 hours after infection. PCR products: flox, 812 bp; null, 777 bp. (B) Western Blot analysis of the different genotypes to monitor loss of endogenous RTEL1 96 hours after Cre infection. (C) Representative images of the telomere phenotypes observed in Figure 1A. Images show a representative metaphase telomere FISH of the indicated genotypes from SV40-LT (T1 and T2) and primary (C3 and C4) cells. Telomere loss, indicated with yellow arrows; telomere fragility, indicated with red arrows; telomere length heterogeneity, indicated with white arrows. (D) Quantification of T-circle formation in cells from the indicated. Error bars indicate ± SD from three independent experiments. (E) Telomere length analysis of cells from the indicated genotypes.

To determine whether telomerase prevents telomere fusions and DDR induction at telomeres in *Rtel1*^*−/−*^ cells, *Rtel1*^*f/f*^ conditional mice were crossed with early generation *Terc*^*+/−*^ mice, which lack the RNA component of telomerase (*Terc*). Mouse adult ear fibroblast (MAF) cell lines were derived from aged matched *Rtel1*^*f/f*^*Terc*^*+/+*^ and *Rtel1*^*f/f*^*Terc*^*−/−*^ sibling mice. These cells carry *Rtel1* floxed alleles, which allow the conditional deletion of the *Rtel1* gene by Cre-mediated recombination (Sarek et al., 2015; Figures S1A and S1B). In contrast to *Trf2*^*−/−*^ cells, which exhibit extensive telomere fusions, no fusions were observed following Cre-mediated inactivation of RTEL1, irrespective of the status of telomerase (Figures 1A and 1B). These data establish that removing telomerase does not lead to telomere fusions in the absence of RTEL1.

Metaphase spreads from *Rtel1*-deficient telomerase positive and negative cells were also scored for the presence of telomere loss, telomere fragility, telomere length heterogeneity, and telomere circles (TCs), which are hallmarks of telomere dysfunction observed following loss of RTEL1 (Figure 1C). To our surprise, the rapid accumulation of dysfunctional telomeres following conditional *Rtel1* inactivation in telomerase positive cells was largely absent in telomerase negative *Terc*^*−/−*^ cells (Figures S1C and 1D, 1E, and 1F). This result was confirmed in MAFs immortalized by SV40-LT (T1 and T2, and 2 other pairs not shown), as well as in two independently derived sets of primary MAFs (C3 and C4, and C5 and C6). Immortalized *Terc*^*−/−*^ cells (T2) have a basal level of telomere loss even in the presence of RTEL1, but importantly this is not further increased upon RTEL1 inactivation. Moreover, primary *Terc*^*−/−*^ cells (C3 and C4, and C5 and C6), which do not show telomere loss under basal conditions, do not accumulate dysfunctional telomeres upon RTEL1 depletion. In agreement, TCs, which accumulate in RTEL1-deficient cells concomitant with telomere shortening and loss, were induced in *Rtel1*^*f/f*^*Terc*^*+/+*^ cells but this accumulation was largely reduced in *Rtel1*^*f/f*^*Terc*^*−/−*^ cells (Figures 1G and S1D).

### Deletion of *Terc* or *Tert* Prevents Telomere Dysfunction and Suppresses SLX4 Recruitment to Telomeres

**Figure 2:**
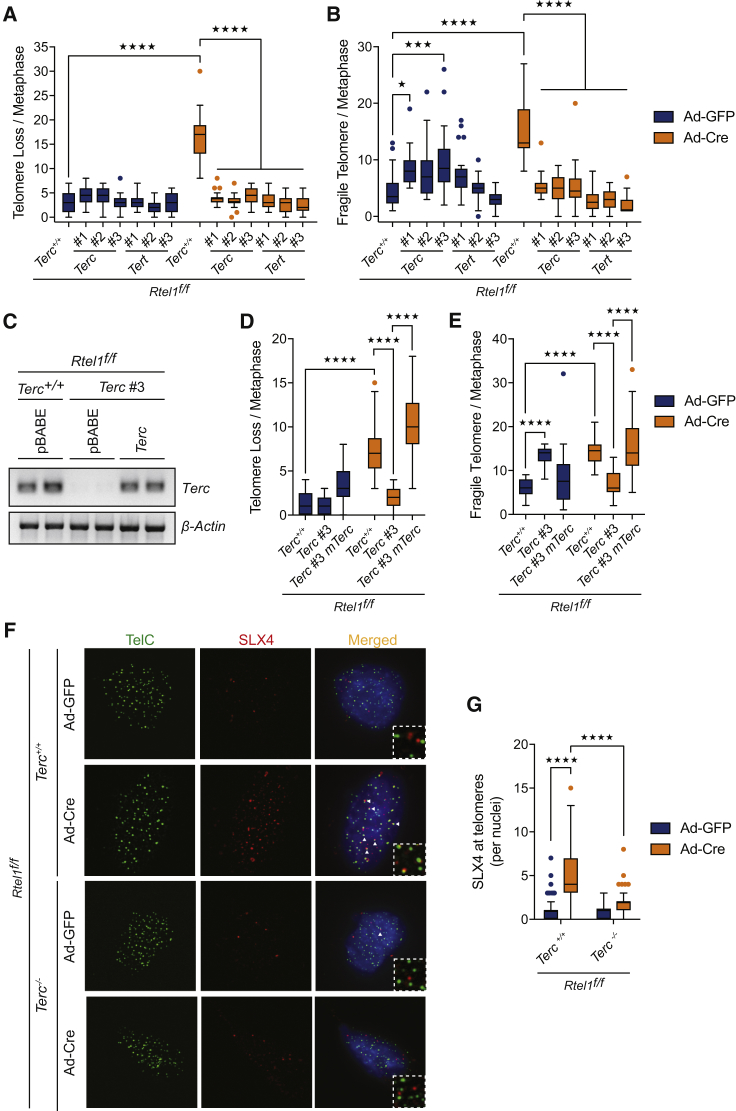
Deletion of *Terc* or *Tert* Prevents Telomere Dysfunction and Suppresses SLX4 Recruitment to Telomeres (A and B) Quantification of telomere loss (A) and telomere fragility (B) per metaphase in cells of the indicated genotype 96 hr after Ad-GFP or Ad-Cre infection. Boxplots represent the quantification from at least 30 metaphases from a representative experiment (^∗^p < 0.05; ^∗∗∗^p < 0.001; ^∗∗∗∗^p < 0.0001; two-way ANOVA). (C) Gel image showing expression of *Terc* in the different genotypes compared to *β-Actin*. (D and E) Quantification of telomere loss (D) and telomere fragility (E) per metaphase in cells of the indicated genotype 96 hr after Ad-GFP or Ad-Cre infection. Boxplots represent the quantification from at least 30 metaphases from a representative experiment (^∗∗∗∗^p < 0.0001; two-way ANOVA). (F) Immunofluorescence (IF)-FISH for TelC (telomeres-green) and SLX4 (red) in cells of the indicated genotypes. Merged image shows TelC, SLX4, and DAPI. Arrows indicate overlapping TelC and SLX4 signals. (G) Quantification of the number of SLX4 foci coincident with telomeres per nuclei of the indicated genotypes. Boxplots represent the quantification from at least 100 nuclei from a representative experiment (^∗∗∗∗^p < 0.0001; two-way ANOVA). See also Figures S2.

**Figure S2:**
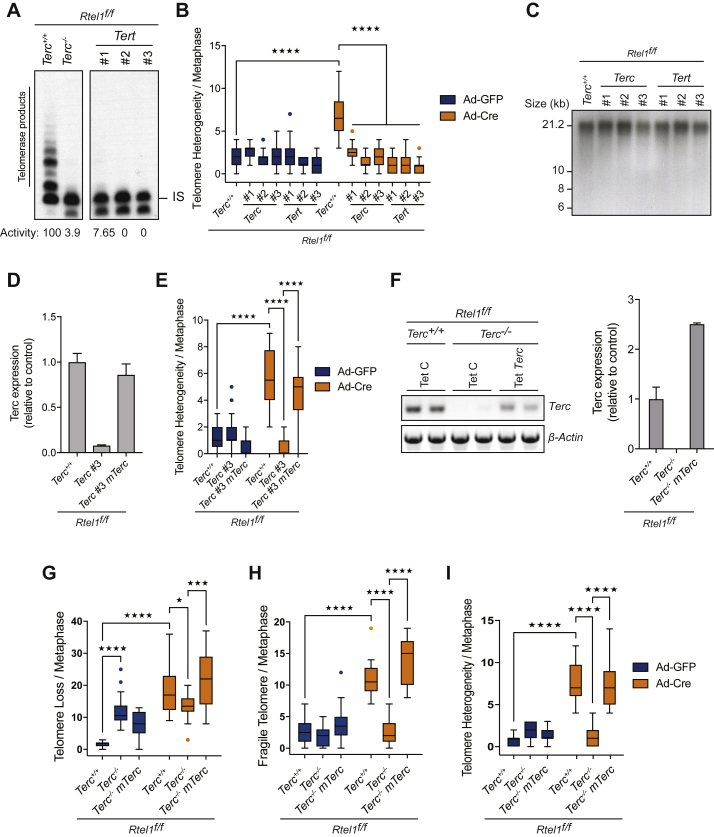
Deletion of *Terc* or *Tert* Prevents Telomere Dysfunction and Suppresses SLX4 Recruitment to Telomeres, Related to Figure 2 (A) Analysis of telomerase activity determined by TRAP assay in the different indicated clones. Telomerase activity was measured relative to the control and normalized to the internal standard (IS). (B) Quantification of telomere length heterogeneity per metaphase 96 hours after Ad-GFP or Ad-Cre infection. Boxplots represent the quantification from at least 30 metaphases from a representative experiment (^∗∗∗∗^p < 0.0001; two-way ANOVA). (C) Telomere length analysis of cells from the indicated genotypes. (D) Quantitative reverse transcription polymerase chain reaction (qRT-PCR) analysis of *Terc* gene. Data are means ± SD normalized to the expression β–Actin and relative to Rtel1^f/f^Terc^+/+^ cells. (E) Quantification of telomere length heterogeneity per metaphase 96 hours after Ad-GFP or Ad-Cre infection. Boxplots represent the quantification from at least 30 metaphases from a representative experiment (^∗∗∗∗^p < 0.0001; two-way ANOVA). (F) Gel image showing expression of *Terc* in the different genotypes compared to β–Actin. On the right, quantitative reverse transcription polymerase chain reaction (qRT-PCR) analysis of *Terc* gene. Data are means ± SD normalized to the expression *β–Actin* and relative to Rtel1^f/f^Terc^+/+^ cells. (G and H) Quantification of telomere loss (G), telomere fragility (H), and telomere length heterogeneity (I) per metaphase 96 hours after Ad-GFP or Ad-Cre infection. Boxplots represent the quantification from at least 30 metaphases from a representative experiment (^∗∗∗∗^p < 0.0001; two-way ANOVA).

To determine whether inactivation of other telomerase components is capable of suppressing telomere dysfunction associated with loss of RTEL1, we generated CRISPR knockouts for both *Terc* and *Tert* genes in conditional *Rtel1*^*f/f*^ MEFs. CRISPR induced deletions in *Terc* and *Tert* were analyzed by DNA sequence and loss of telomerase activity was confirmed using an established Telomeric Repeat Amplification Protocol (TRAP) (Tables S1; Figure S2A). In agreement with our previous results in MAF cells, MEFs lacking *Terc* or *Tert* did not show *Rtel1* telomeric dysfunction after Cre infection when assessed for telomeric loss, telomeric fragility, or telomeric length heterogeneity (Figure 2A, 2B, and S2B). The fact that telomere lengths are comparable between *Terc*^+/+^, *Terc*^−/−^, and the CRISPR cell lines excludes the possibility that telomere shortening in the absence of telomerase is responsible for rescuing the deleterious effects of RTEL1 deficiency (Figures S1E and S2C).

To further confirm that the lack of telomeric dysfunction in the *Rtel1*^*−/−*^*Terc*^*−/−*^ and *Rtel1*^*−/−*^ *Terc* CRISPR cells is due to *Terc* inactivation, we re-expressed Terc RNA in these cells (Figure 2C, S2D, and S2F). Restoring Terc RNA expression to *Rtel1*^*−/−*^*Terc*^*−/−*^ cells or to *Rtel1*^*−/−*^ *Terc* CRISPR cells through expression of a Terc transgene resulted in the reappearance of telomeric loss, telomeric fragility, and telomeric length heterogeneity following *Rtel1* inactivation (Figures 2D, 2E, S2E, S2G, S2H, and S2I).

Our observations raised the possibility that telomerase is important for the accumulation of SLX1/4 at telomeres in RTEL1-null cells, which processes persistent t-loops (Vannier et al., 2012). Indeed, telomerase positive *Terc*^*+/+*^ cells lacking RTEL1 showed a 5-fold increase in SLX4 foci that overlap with telomeres, when compared to *Rtel1*-proficient cells (Figures 2F and 2G). Conversely, the accumulation of SLX4 at telomeres was suppressed in telomerase negative *Rtel1*^*−/−*^*Terc*^*−/−*^ cells. Hence, telomerase is necessary for the accumulation of the SLX1/4 nuclease complex at *Rtel1*-deficient telomeres.

### *Terc* Depletion Does Not Rescue the Replication Defects Associated with *Rtel1* Dysfunction

**Figure 3:**
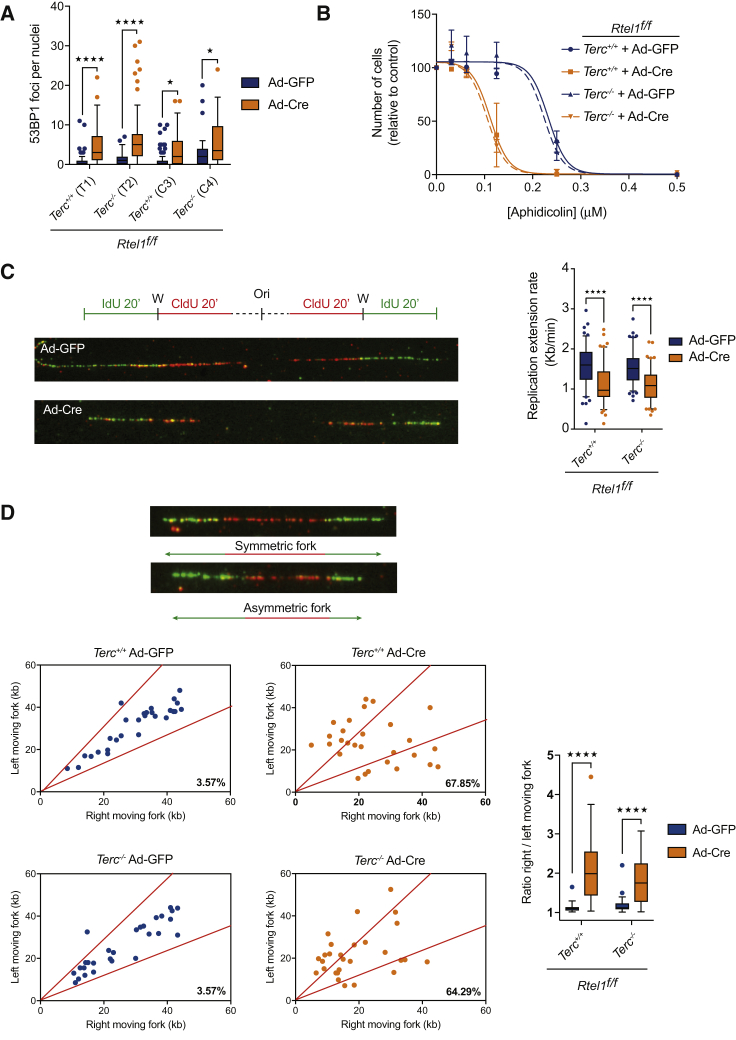
*Terc* Depletion Does Not Rescue the Replication Defects Associated with *Rtel1* Dysfunction (A) Quantification of non-telomere-associated 53BP1 foci per nuclei in the different genotypes. Boxplots represent the quantification from at least 150 nuclei from a representative experiment (^∗^p < 0.05; ^∗∗∗∗^p < 0.0001; two-way ANOVA). (B) Sensitivity of cells of the indicated genotype to increasing doses of Aphidicolin. Error bars, ±SD from three independent experiments. (C) Replication fork dynamics in cells of the indicated genotypes pulse-labeled with chlorodeoxyuridine (CldU) followed by iododeoxyuridine (IdU) and subjected to DNA combing. Images show representative fibers from Ad-GFP or Ad-Cre treatments. 150 fibers were measured per genotype, and replication fork speed was measured in kb/min (^∗∗∗∗^p < 0.0001; two-way ANOVA). (D) Representative images of symmetric and asymmetric forks. Quantification of the degree of fork asymmetry in the different genotypes. 30 fibers were measured per genotype (^∗∗∗∗^p < 0.0001; two-way ANOVA). Percentage represents the amount of asymmetric fibers relative to the total amount of measured.

RTEL1’s role in cells is not limited to telomere maintenance but also extends to global DNA replication (Vannier et al., 2013); we asked whether telomerase also suppresses the non-telomeric phenotypes associated with *Rtel1* deficiency. To this end, we examined the levels of 53BP1 foci as a readout of spontaneous DNA damage and assessed replication dynamics by aphidicolin treatment and DNA combing. Non-telomere-associated 53BP1 foci were elevated in both immortalized and primary *Rtel1*^*−/−*^*Terc*^*+/+*^ and *Rtel1*^*−/−*^*Terc*^*−/−*^ cells, suggesting that induction of spontaneous DNA damage following loss of RTEL1 occurs independently of telomerase status (Figure 3A). Consistent with a previous report, *Rtel1*^*−/−*^ *Terc*^*+/+*^ cells exhibited sensitivity to aphidicolin treatment when compared to *Rtel1*-proficient cells (Uringa et al., 2012; Figure 3B). Importantly, *Rtel1*^*−/−*^ *Terc*^*−/−*^ cells displayed a similar level of aphidicolin sensitivity to telomerase positive *Rtel1*^*−/−*^ *Terc*^*+/+*^ cells, suggesting that *Terc* deletion does not influence the global replication defects associated with *Rtel1* inactivation. In support of this conclusion, DNA combing revealed that the reduced replication fork extension rates and increased fork asymmetry observed following *Rtel1* inactivation occur irrespective of telomerase status: *Rtel1*^*−/−*^ *Terc*^*+/+*^ and *Rtel1*^*−/−*^*Terc*^*−/−*^ cells both exhibited reduced replication fork speeds when compared to *Rtel1*-proficient cells (*Rtel1*^*+/+*^ 1.597 kb/min versus *Rtel1*^*−/−*^*Terc*^*+/+*^ 1.095 kb/min and *Rtel1*^*−/−*^*Terc*^*−/−*^ 1.085 kb/min) (Figure 3C). Similarly, the accumulation of asymmetric replication forks observed in *Rtel1*-deficient cells, which reflect increased fork stalling and/or collapse, occurs irrespective of telomerase status (*Rtel1*^*+/+*^ cells 3.57% asymmetric DNA tracts versus *Rtel1*^*−/−*^*Terc*^*+/+*^ 67.85% and *Rtel1*^*−/−*^*Terc*^*−/−*^ 64.29%; Figure 3D). These results establish that suppression of the *Rtel1* phenotype by telomerase inactivation does not extend to the role of RTEL1 during global DNA replication.

### Stabilization of DNA Secondary Structures Leads to Aberrant Accumulation of Telomerase at Telomeres

**Figure S3:**
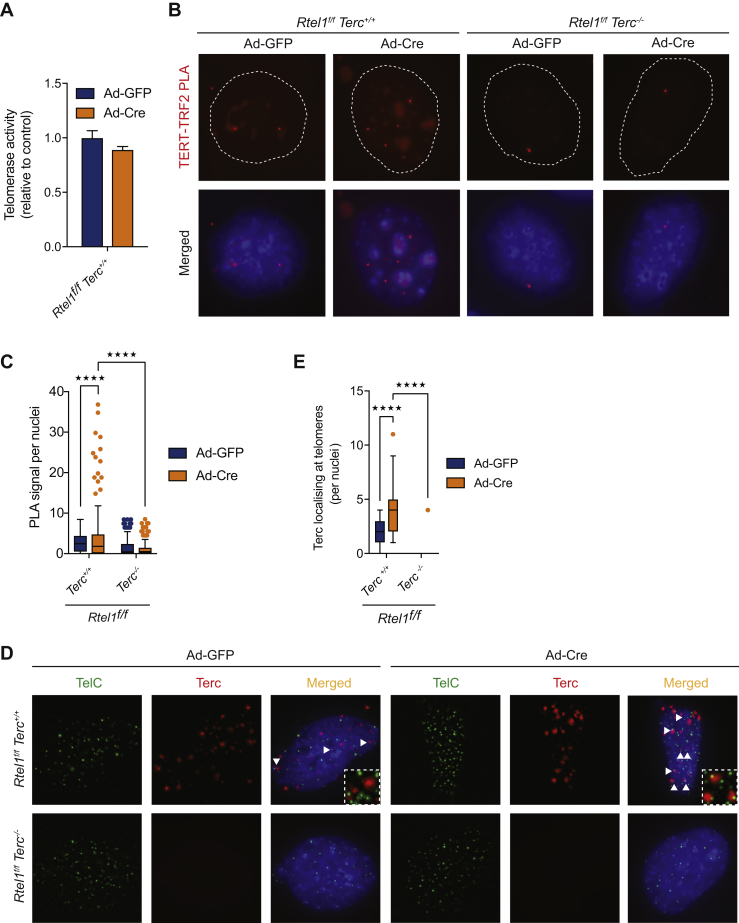
Stabilization of DNA Secondary Structures Leads to Aberrant Accumulation of Telomerase at Telomeres, Related to Figure 4 (A) Analysis of telomerase activity determined by TRAP assay in the different indicated genotypes. Error bars indicate ± SD from two independent experiments. (B and C) Quantification of the interaction between TERT and TRF2 as determined by *in situ* PLA assay in the cells indicated. Data represents quantification from at least 150 nuclei from a representative experiment (^∗∗∗∗^p < 0.0001; two-way ANOVA). Dashed lines indicate nucleus (as determined by DAPI in blue). (D and E) Representative images and quantification of the localisation of Terc RNA (red) at telomeres (TelC-Green) as determined by *in situ* RNAscope assay coupled to telomere FISH in cells of the indicated genotype. Arrows indicate colocalization between Terc RNA and TelC (telomere). Data represents quantification from at least 150 nuclei from a representative experiment (^∗∗∗∗^p < 0.0001; two-way ANOVA).

We considered two possibilities that could account for why the presence of telomerase could result in telomere dysfunction in RTEL1-deficient cells: (1) telomerase is corrupted by loss of *Rtel1* and this interferes with telomere biogenesis, or (2) telomerase inappropriately binds to and stabilizes DNA secondary structures at the telomere that are normally removed by RTEL1. The first of these possibilities is unlikely, as telomerase activity is comparable between *Rtel1*-proficient and -deficient cells (Figure S3A). We therefore focused our attention on the second hypothesis and sought to determine whether telomerase abnormally engages with telomeres when telomeric DNA secondary structures are not properly dismantled.

**Figure 4:**
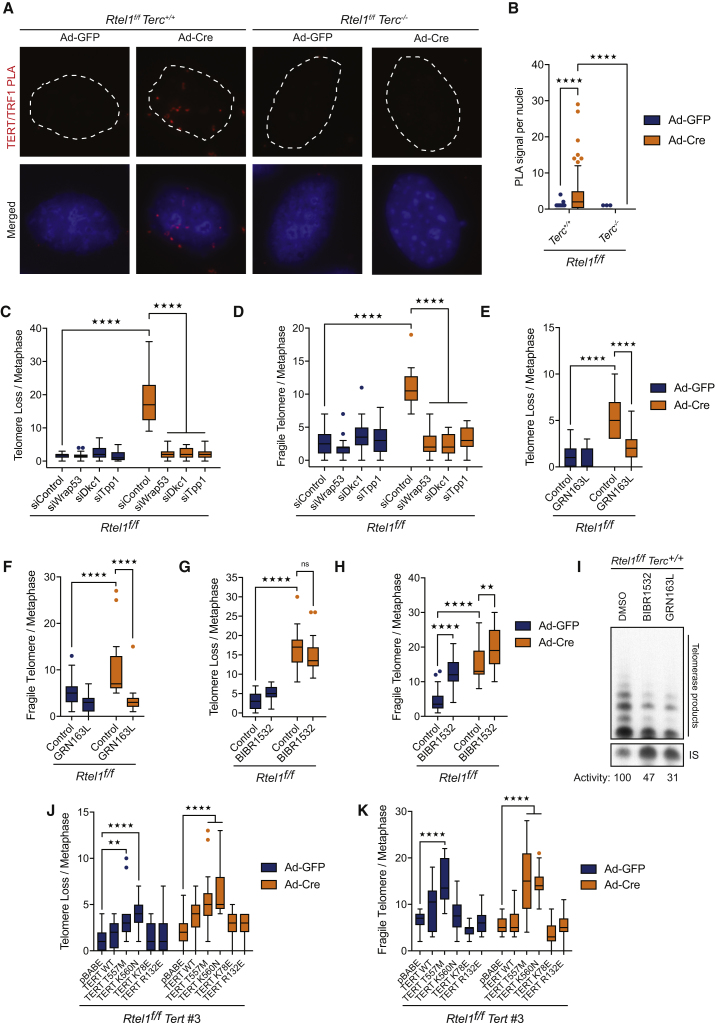
Stabilization of DNA Secondary Structures Leads to Aberrant Accumulation of Telomerase at Telomeres (A and B) Representative images (A) and quantification of the frequency (B) of interaction between TERT and TRF1 as determined by *in situ* PLA assay in cells of the indicated genotype. Dashed lines indicate nucleus (as determined by DAPI in blue). Data represent quantification from at least 150 nuclei from a representative experiment (^∗∗∗∗^p < 0.0001; two-way ANOVA). (C and D) Quantification of telomere loss (C) and telomere fragility (D) per metaphase 96 hr after Ad-GFP or Ad-Cre infection in cells of the indicated genotype. Boxplots represent the quantification from at least 30 metaphases from a representative experiment (^∗∗∗∗^p < 0.0001; two-way ANOVA). (E–H) Quantification of telomere loss (E and G) and telomere fragility (F and H) per metaphase 96 hr after Ad-GFP or Ad-Cre infection in cells treated with GRN163L (E and F) or BIBR1532 (G and H). Representative images of telomere FISH on metaphases are shown in Figure S5D. Boxplots represent the quantification from at least 30 metaphases from a representative experiment (^∗∗^p < 0.01; ^∗∗∗∗^p < 0.0001; two-way ANOVA). (I) Analysis of telomerase activity determined by TRAP assay on cells of the indicated genotype treated with BIBR1532 or GRN162L. Telomerase activity was measured relative to the control and normalized to the internal standard (IS). (J and K) Quantification of telomere loss (J) and telomere fragility (K) per metaphase 96 hr after Ad-GFP or Ad-Cre infection in cells of the indicated genotype. Representative images of telomere FISH on metaphases are shown in Figure S5M. Boxplots represent the quantification from at least 30 metaphases from a representative experiment (^∗∗^p < 0.01; ^∗∗∗∗^p < 0.0001; two-way ANOVA). See also Figures S3 and S4.

A recent study has defined two distinct populations of telomerase transiently engaging with telomeres (Schmidt et al., 2016), which are believed to reflect: (1) short-lived but frequent interactions with TPP1 that are not associated with the 3′ overhang at the chromosome end, and (2) long static interactions with the 3′ overhang that are productive for telomere extension but are very rare. To examine telomerase recruitment to telomeres in *Rtel1*-proficient and -deficient cells, we conducted proximity ligation assays (PLAs) to detect close proximity interactions between the TERT subunit of telomerase and the Shelterin protein TRF1. In *Rtel1*-proficient cells, PLA between endogenous TERT and TRF1 is an infrequent event, consistent with the low abundance and transient nature of telomerase binding to telomeres in wild-type cells (Figures 4A and 4B). In contrast, a 4-fold increase in PLA signal for TERT and TRF1 was detected in *Rtel1*-deficient cells when compared to controls cells (Figures 4A and 4B). We also detected a robust PLA signal between TERT and TRF2 in *Rtel1*-deficient cells, but not in control cells (Figures S3B and S3C). A significant increase in Terc RNA coincident with telomeres was observed by RNAscope in *Rtel1*-deficient cells, but not in *Rtel1*^*−/−*^*Terc*^*−/−*^ cells (Figures S3D and S3E). These data reveal that telomerase binds abnormally to telomeres in the absence of RTEL1.

**Figure S4:**
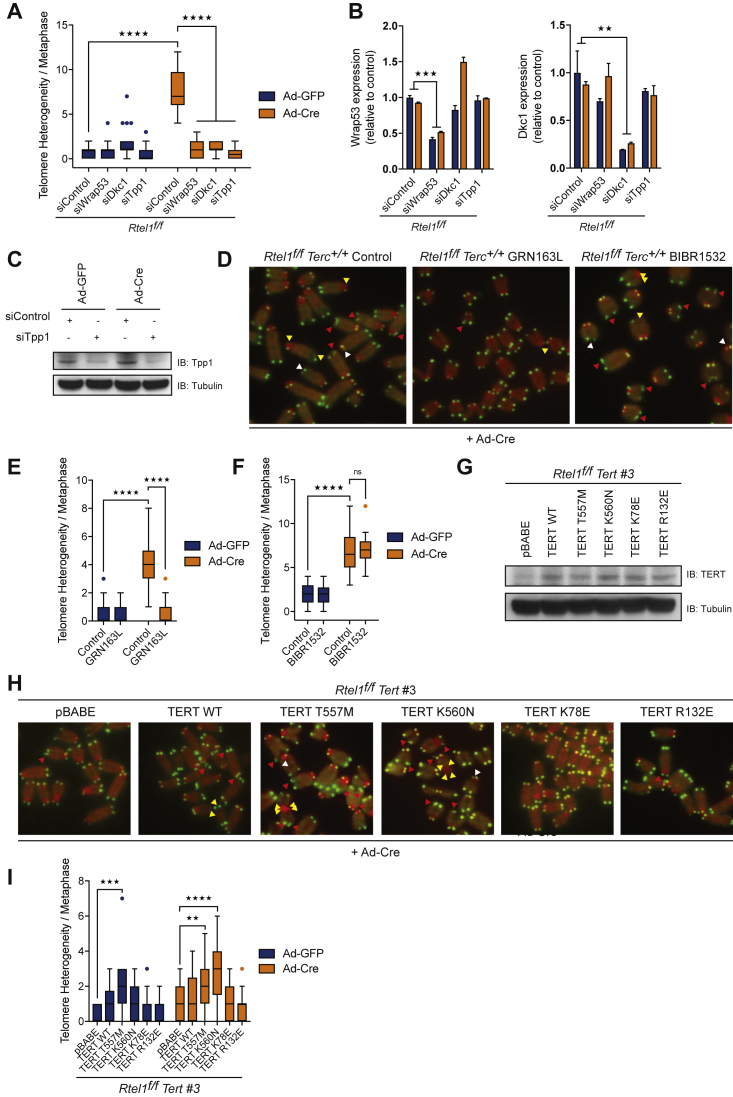
Stabilization of DNA Secondary Structures Leads to Aberrant Accumulation of Telomerase at Telomeres, Related to Figure 4 (A) Quantification of telomere length heterogeneity per metaphase 96 hours after Ad-GFP or Ad-Cre infection. Boxplots represent the quantification from at least 30 metaphases from a representative experiment (^∗∗∗∗^p < 0.0001; two-way ANOVA). (B) qRT-PCR analysis of the indicated genes. Data are means ± SD normalized to the expression β–Actin and relative to control. (^∗∗^p < 0.01^∗∗∗^p < 0.001; two-way ANOVA). (C) Western Blot analysis of the different genotypes to monitor loss of endogenous TPP1 96 hours after Cre infection. (D) Images show a representative metaphase telomere FISH of the indicated drug treatments. Telomere loss, indicated with yellow arrows; telomere fragility, indicated with red arrows; telomere length heterogeneity, indicated with white arrows. (E and F) Quantification of telomere length heterogeneity per metaphase 96 hours after Ad-GFP or Ad-Cre infection in cells treated with GRN163L (E) or BIBR1532 (F). Boxplots represent the quantification from at least 30 metaphases from a representative experiment (^∗∗∗∗^p < 0.0001; two-way ANOVA). (G) Western Blot analysis of the different genotypes to monitor expression of TERT. (H) Images show a representative metaphase telomere FISH of the indicated genotypes. Telomere loss, indicated with yellow arrows; telomere fragility, indicated with red arrows; telomere length heterogeneity, indicated with white arrows. (I) Quantification of telomere length heterogeneity per metaphase 96 hours after Ad-GFP or Ad-Cre infection. Boxplots represent the quantification from at least 30 metaphases from a representative experiment (^∗∗∗∗^p < 0.0001; two-way ANOVA).

Since telomerase recruitment to telomeres requires the shelterin component TPP1, as well as DKC1 and TCAB1 (*Wrap53* in mice) (Zhong et al., 2012), we asked what would happen to *Rtel1*-deficient telomeres if we blocked telomerase recruitment. Strikingly, downregulation of *Tpp1*, *Dkc1*, or *Wrap53* (Figures S4B and S4C) abolished the rapid accumulation of dysfunctional telomeres following *Rtel1* inactivation (Figures 4C, 4D, and S4A). Furthermore, treating *Rtel1*^*f/f*^ cells with GRN163L, which inhibits telomerase recruitment to telomeres (Asai et al., 2003, Herbert et al., 2005), blocked the appearance of dysfunctional telomeres in these cells (Figures 4E, 4F, S4D, and S4E). In contrast, treatment of *Rtel1*^*−/−*^ cells with BIBR1532, which directly inhibits telomerase enzymatic activity without affecting its recruitment to telomeres (Pascolo et al., 2002), failed to prevent telomeric catastrophe (Figures 4G, 4H, 4I, S4D, and S4F). These results were further confirmed by re-expressing WT TERT or different TERT mutants in *Rtel1*^*f/f*^ *Tert* CRISPR cells (Figure S4G). *Rtel1*-null cells expressing TERT mutants with reduced/abolished catalytic activity (TERT T557M and TERT K560N mutants [Gramatges et al., 2013]) presented with dysfunctional telomeres (Figures 4J, 4K, S4H, and S4I). In contrast, cells expressing TERT mutants defective for recruitment to telomeres (TERT K78E and TERT R132E [Schmidt et al., 2014]) were devoid of telomere dysfunction. Hence, telomere dysfunction in *Rtel1*-deficient cells depends on telomerase recruitment to telomeres, but not its catalytic activity.

### *Rtel1* Telomeric Dysfunction Is Rescued by Blocking Replication Fork Reversal

**Figure 5:**
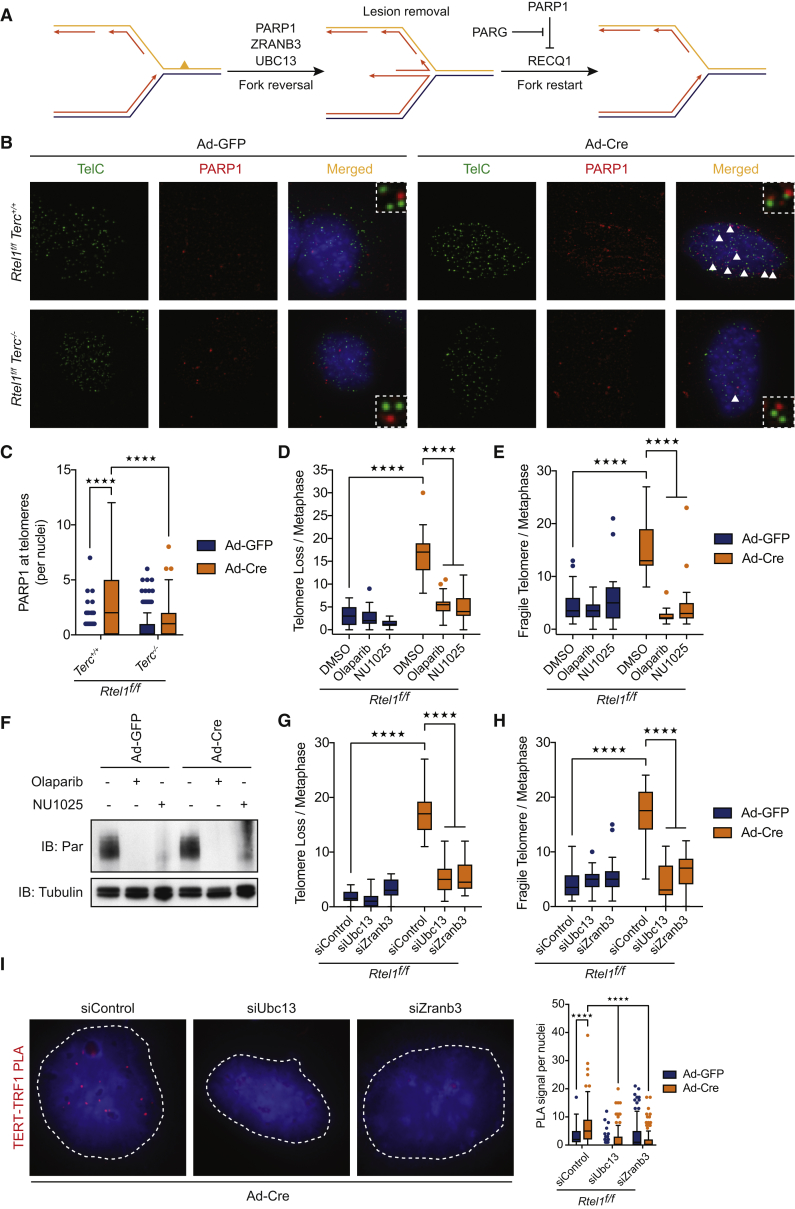
*Rtel1* Telomeric Dysfunction Is Rescued by Blocking Replication Fork Reversal (A) Schematic of the process of replication fork reversal and the different genes involved in fork reversal and fork restart. (B and C) Representative images (B) and quantification of the frequency (C) of PARP1 foci (red) coincident with telomeres (TelC, green) in cells of the indicated genotypes. Arrows indicate TelC-PARP1 colocalization events. Boxplots represent the quantification from at least 150 nuclei from a representative experiment (^∗∗∗∗^p < 0.0001; two-way ANOVA). (D and E) Quantification of telomere loss (D) and telomere fragility (E) per metaphase in cells of the indicated genotype 96 hr after Ad-GFP or Ad-Cre infection. Boxplots represent the quantification from at least 30 metaphases from a representative experiment (^∗∗∗∗^p < 0.0001; two-way ANOVA). (F) Western blot analysis showing PARylated proteins in cells subject to the indicated PARP inhibitor treatments (5 μM Olaparib and 10 μM NU1025). (G and H) Quantification of telomere loss (G) and telomere fragility (H) per metaphase in cells of the indicated genotype 96 hr after Ad-GFP or Ad-Cre infection. Representative images of telomere FISH on metaphases are shown in Figure S7A. Boxplots represent the quantification from at least 30 metaphases from a representative experiment (^∗∗∗∗^p < 0.0001; two-way ANOVA). (I) Representative images and quantification of the frequency of interaction between TERT and TRF1 as determined by *in situ* PLA assay in *Rtel1*-deficient cells subject to the indicated siRNA treatment. Data represent quantification from at least 150 nuclei from a representative experiment (^∗∗∗∗^p < 0.0001; two-way ANOVA). Dashed lines indicate nucleus (as determined by DAPI in blue). See also Figures S3.

A key insight into the mechanism by which telomerase drives telomere dysfunction in the absence of RTEL1 came from analysis of the DNA damage response at telomeres. Poly(ADP-ribose) polymerase (PARP) is an NAD-dependent enzyme that catalyzes PARylation of DNA replication, repair, and chromatin proteins in response to a wide range of DNA lesions, stresses, and impediments to the replication fork. Immunostaining of telomerase-positive *Rtel1*^*−/−*^*Terc*^*+/+*^ cells revealed an enhanced accumulation of PARP1 foci coincident with telomeres, which were reduced in telomerase-negative *Rtel1*^*−/−*^*Terc*^*−/−*^ cells (Figures 5B and 5C). This observation led us to consider the possibility that the accumulation of dysfunctional telomeres could be linked to replication fork reversal, which requires PARP1.

Replication fork reversal occurs when the advancing replisome encounters DNA lesions or obstacles in DNA (reviewed in Neelsen and Lopes, 2015) (Figure 5A). This a highly regulated process during which the replication fork is remodeled into a regressed chicken-foot (4-way junction) structure by annealing of newly synthesized strands and re-annealing of parental strands (visualized in Sogo et al., 2002). Fork reversal can facilitate fork stabilization and provide time to either remove the offending lesion/obstacle or initiate lesion bypass by polymerase switching or re-priming (Mijic et al., 2017, Ray Chaudhuri et al., 2012, Vujanovic et al., 2017, Zellweger et al., 2015). However, unscheduled fork reversal can expose the fork to aberrant nucleolytic attack, which may lead to genome instability (Couch et al., 2013, Dungrawala et al., 2017, Lemaçon et al., 2017, Mijic et al., 2017, Neelsen et al., 2013, Taglialatela et al., 2017, Thangavel et al., 2015). Hence, depending on context, replication fork reversal can provide a means to deal with replication stress but if uncontrolled can result in pathological consequences for the genome.

PARP1 is essential for fork reversal (Ray Chaudhuri et al., 2012) and acts by inhibiting the fork restart activity of the RECQ1 helicase (Berti et al., 2013). Fork reversal also requires UBC13-dependent poly-ubiquitination of PCNA on lysine 164, which recruits the fork reversal enzymes ZRANB3 and SMARCAL1 (Bétous et al., 2012, Bétous et al., 2013, Ciccia et al., 2012, Vujanovic et al., 2017, Weston et al., 2012, Zellweger et al., 2015). RAD51 is also essential for fork reversal and along with BRCA2 acts to protect newly synthesized DNA at the fork from promiscuous nucleolytic degradation by MRE11 (Schlacher et al., 2011). Once the lesion/obstacle has been removed, fork restart is achieved by reactivation of RECQ1 activity by PARG-dependent removal of PAR (Figure 5A) (Berti et al., 2013, Ray Chaudhuri et al., 2015). As such, knockdown/inhibition of PARP1, RAD51, UBC13, ZRANB3, or SMARCAL1 blocks fork reversal, whereas elimination of RECQ1 or PARG prevents fork restart *in vivo*.

**Figure S5:**
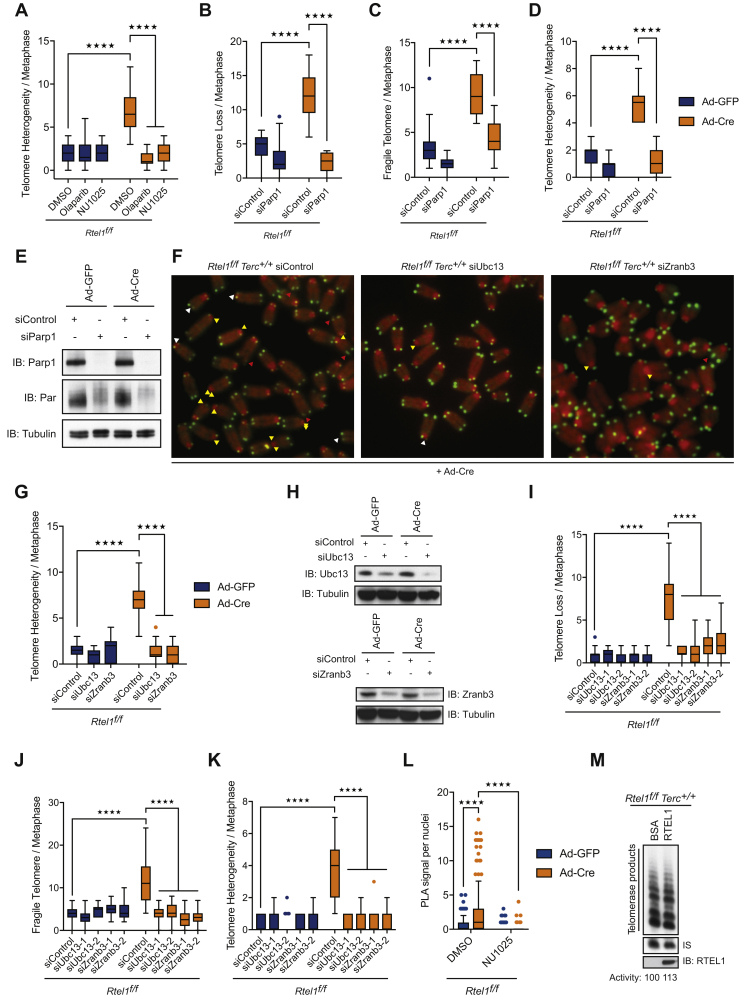
*Rtel1* Telomeric Dysfunction Is Rescued by Blocking Replication Fork Reversal, Related to Figure 5 (A) Quantification of telomere length heterogeneity per metaphase 96 hours after Ad-GFP or Ad-Cre infection. Boxplots represent the quantification from at least 30 metaphases from a representative experiment (^∗∗∗∗^p < 0.0001; two-way ANOVA). (B and D) Quantification of telomere loss (B), telomere fragility (C), and telomere length heterogeneity (D) per metaphase 96 hours after Ad-GFP or Ad-Cre infection. Boxplots represent the quantification from at least 30 metaphases from a representative experiment (^∗∗∗∗^p < 0.0001; two-way ANOVA). (E) Western Blot analysis of the different genotypes to monitor loss of endogenous PARP1 96 hours after Cre infection, as well as total PARylated proteins. (F) Images show a representative metaphase telomere FISH of the indicated genotypes. Telomere loss, indicated with yellow arrows; telomere fragility, indicated with red arrows; telomere length heterogeneity, indicated with white arrows. (G) Quantification of telomere length heterogeneity per metaphase 96 hours after Ad-GFP or Ad-Cre infection. Boxplots represent the quantification from at least 30 metaphases from a representative experiment (^∗∗∗∗^p < 0.0001; two-way ANOVA). (H) Western Blot analysis of the different genotypes to monitor loss of endogenous UBC13 (left) and ZRANB3 (right) 96 hours after Cre infection. (I and K) Quantification of telomere loss (I), telomere fragility (J), and telomere length heterogeneity (K) per metaphase 96 hours after Ad-GFP or Ad-Cre infection. Boxplots represent the quantification from at least 30 metaphases from a representative experiment (^∗∗∗∗^p < 0.0001; two-way ANOVA). (L) Quantification of the interaction between TERT and TRF1 as determined by *in situ* PLA assay in the different indicated treatments. Data represents quantification from at least 150 nuclei from a representative experiment (^∗∗∗∗^p < 0.0001; two-way ANOVA). (M) Analysis of telomerase activity determined by TRAP assay in the presence of BSA or recombinant RTEL1. Telomerase activity was measured relative to the control and normalized to the internal standard (IS).

We considered the possibility that the enhanced PARP1 foci seen at *Rtel1*-deficient telomeres might reflect the activation of fork reversal in response to impediments within the telomere, such as persistent t-loops or telomeric G4-DNA structures. With this hypothesis in mind, we first asked what would happen to telomeres in *Rtel1*-null cells if we blocked PARP1 activity. Strikingly, treatment of *Rtel1*-deficient cells with two different inhibitors of PARP1 (Olaparib or NU1025) abolished telomere loss, telomere fragility, and telomeric length heterogeneity in these cells (Figures 5D, 5E, and S5A). A decrease in PARylated proteins confirmed the efficiency of both PARP inhibitors (Figure 5F). Downregulation of PARP1 by small interfering RNA (siRNA) also suppressed the *Rtel1*-null phenotype (Figures S5B, S5C, S5D, and S5E). Since PARP1 plays multiple roles in cells, it is not possible to conclude, based on this result alone, that the suppression of telomere catastrophe in *Rtel1*-deficient cells is due to its role in fork reversal. We therefore examined the impact of downregulating UBC13 and ZRANB3, which are also essential for fork reversal (Figure 5A). Similar to the results with PARP1 inhibition, downregulation of either UBC13 or ZRANB3 abolished the appearance of telomere dysfunction in *Rtel1*-null cells (Figures 5G, 5H, S5F, and S5G). siRNA knockdown efficiency was assessed by western blotting (Figure S5H). To confirm the specificity of UBC13 and ZRANB3 siRNAs, we repeated these experiments using individual siRNAs against both genes and obtained the same result (Figures S5I, S5J, and S5K). Collectively, these results support a role for fork reversal in driving telomere catastrophe in *Rtel1*-deficient cells.

Since replication fork reversal within the telomere is predicted to create a chicken-foot structure with a telomeric 3′ overhang on the regressed single DSB end (Figure 5A), binding of this structure by telomerase could explain the aberrant accumulation of telomerase at *Rtel1*-deficient telomeres. We reasoned that if telomerase binds to the regressed single DSB end of a reversed replication fork in *Rtel1*-null cells, then blocking fork reversal should abolish its aberrant binding to telomeres. Indeed, blocking PARP1 activity with the inhibitor NU1025 or downregulating *Ubc13* or *Zranb3* by siRNA, significantly reduced the interaction between TERT and TRF1 in *Rtel1*-deficient cells, as measured by PLA (Figures 5I and S5L). We considered the possibility that RTEL1 may act to remove telomerase from reversed replication forks within the context of the telomere. However, the ability of telomerase to extend telomeres as measured by TRAP assay was unaffected by the addition of recombinant RTEL1 (Figure S5M), suggesting that this is not the case. Collectively, these results suggest that telomerase inappropriately binds to reversed replication forks and blocking fork reversal is sufficient to suppress telomere catastrophe in *Rtel1*-deficient cells.

### Prevention of Replication Fork Restart Mimics Telomerase-Induced Telomere Dysfunction

If the aberrant binding of telomerase to reversed replication forks acts to prevent replication fork restart, such a scenario could explain why t-loops are ultimately cleaved by SLX1/4 at *Rtel1*-deficient telomeres, thereby removing the obstacle to the replication fork. If this hypothesis is correct, then blocking replication fork restart should mimic the presence of telomerase, stabilize the reversed fork and restore telomere dysfunction in telomerase-defective cells.

**Figure 6:**
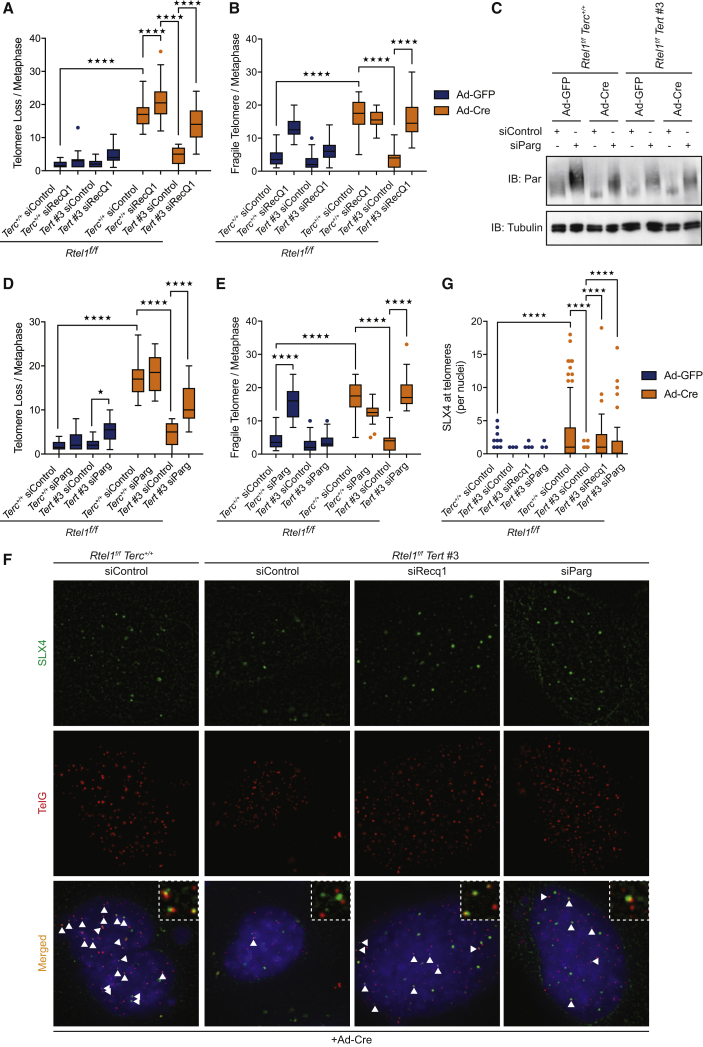
Prevention of Replication Fork Restart Mimics Telomerase-Induced Telomere Dysfunction (A and B) Quantification of telomere loss (A) and telomere fragility (B) per metaphase 96 hr after Ad-GFP or Ad-Cre infection in cells subject to the indicated siRNA treatment. Representative images of telomere FISH on metaphases are shown in Figure S6D. Boxplots represent the quantification from at least 30 metaphases from a representative experiment (^∗∗∗∗^p < 0.0001; two-way ANOVA). (C) Western blot analysis showing PARylated proteins in cells of the indicated genotypes subject to control and Parg siRNA. (D and E) Quantification of telomere loss (D) and telomere fragility (E) per metaphase in cells of the indicated genotype 96 hr after Ad-GFP or Ad-Cre infection. Representative images of telomere FISH on metaphases are shown in Figure S6D. Boxplots represent the quantification from at least 30 metaphases from a representative experiment (^∗∗∗∗^p < 0.0001; two-way ANOVA). (F and G) Representative images (F) and quantification of the frequency (G) of SLX4 (green) colocalizing with TelG (telomeres-red) per nuclei in cells of the indicated genotypes. Arrows indicate SLX4-TelG colocalization events. Boxplots represent the quantification from at least 150 nuclei from a representative experiment (^∗∗∗∗^p < 0.0001; two-way ANOVA). See also Figure S6.

**Figure S6:**
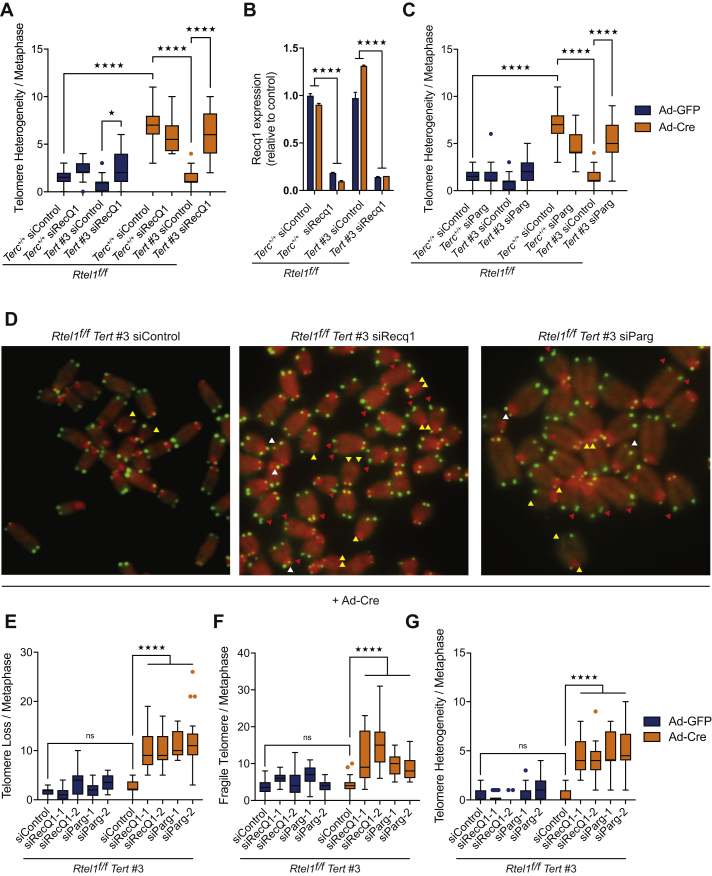
Prevention of Replication Fork Restart Mimics Telomerase-Induced Telomere Dysfunction, Related to Figure 6 (A) Quantification of telomere length heterogeneity per metaphase 96 hours after Ad-GFP or Ad-Cre infection. Boxplots represent the quantification from at least 30 metaphases from a representative experiment (^∗∗∗∗^p < 0.0001; two-way ANOVA). (B) qRT-PCR analysis of *Recq1* gene. Data are means ± SD normalized to the expression *β–Actin* and relative to control. (^∗∗∗∗^p < 0.0001; two-way ANOVA). (C) Quantification of telomere length heterogeneity per metaphase 96 hours after Ad-GFP or Ad-Cre infection. Boxplots represent the quantification from at least 30 metaphases from a representative experiment (^∗∗∗∗^p < 0.0001; two-way ANOVA). (D) Images show a representative metaphase telomere FISH of the indicated genotypes. Telomere loss, indicated with yellow arrows; telomere fragility, indicated with red arrows; telomere length heterogeneity, indicated with white arrows. (E–G) Quantification of telomere loss (E), telomere fragility (F), and telomere length heterogeneity (G) per metaphase 96 hours after Ad-GFP or Ad-Cre infection. Boxplots represent the quantification from at least 30 metaphases from a representative experiment (^∗∗∗∗^p < 0.0001; two-way ANOVA).

To test this possibility, we assessed the consequences of downregulating the replication fork restart activity of the RECQ1 helicase in telomerase-proficient and -deficient cells lacking RTEL1 (*Rtel1*^*−/−*^*Terc*^*+/+*^ and *Rtel1*^*−/−*^*Tert*^*−/−*^ cells, respectively). Strikingly, depletion of RECQ1 by siRNA (Figure S6B) induced telomere dysfunction in *Rtel1*^*−/−*^*Tert*^*−/−*^ cells, which looked phenotypically indistinguishable from *Rtel1*^*−/−*^*Terc*^*+/+*^ cells (Figures 6A, 6B, S6A, S6D, S6E, S6F, and S6G). We also assessed the impact of depleting poly(ADP-ribose) glycohydrolase, PARG, which is essential for overcoming the inhibitory effect of PARylated PARP1 on RECQ1 to allow the replication fork to restart (Berti et al., 2013, Ray Chaudhuri et al., 2015). As expected, downregulating PARG by siRNA resulted in a robust increase in PARylated proteins in cells (Figure 6C). Similar to RECQ1 depletion, downregulation of PARG restored telomere dysfunction in *Rtel1*^*−/−*^ cells lacking telomerase (Figures 6D, 6E, S6C, S6D S6E, S6F, and S6G).

Next, we asked whether blocking replication fork restart in *Rtel1*^*−/−*^*Tert*^*−/−*^ cells leads to recruitment of the SLX1/4 nuclease to telomeres. To this end, we performed SLX4 immunofluorescence coupled with telomere fluorescence *in situ* hybridization (FISH) in *Rtel1*^*−/−*^*Tert*^*−/−*^ cells treated with control, *Recq1*, or *Parg* siRNA. In agreement with previous results, SLX4 is recruited to RTEL1-deficient telomeres but only if telomerase is present. In contrast, depletion of RECQ1 or PARG in *Rtel1*^*−/−*^*Tert*^*−/−*^ cells triggered the recruitment of SLX1/4 to telomeres (Figures 6F and 6G). Collectively, these results raised the possibility that telomerase aberrantly binds to and inhibits replication fork restart, which ultimately results in SLX1/4-dependent telomere catastrophe in the absence of RTEL1.

### Telomerase Binding to Reversed Replication Forks Inhibits Replication at Telomeres

**Figure 7:**
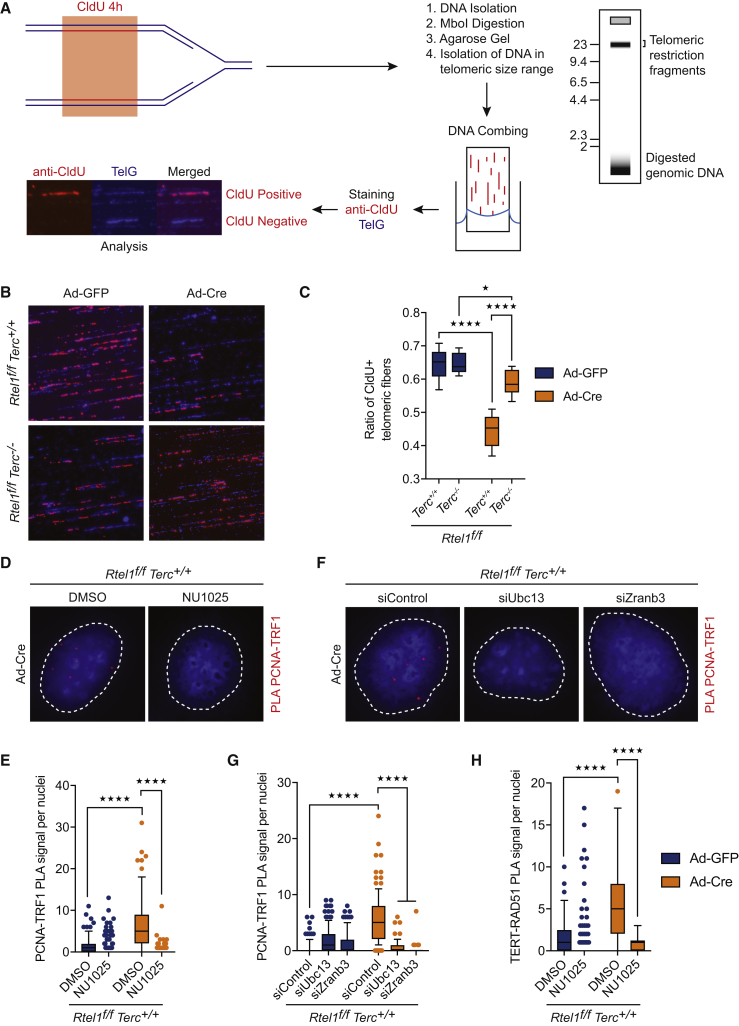
Telomerase Binding to Reversed Replication Forks Inhibits Replication at Telomeres (A) Schematic representation of the SMARD protocol used to monitor replication dynamics at telomeres. The image shows CldU positive and negative telomeric fibers. (B) Representative images of the indicated genotypes showing replication dynamics at telomeres. TelG (blue); CldU (red). (C) Quantification of CldU positive telomeric fibers in cells of the indicated genotype 96 hr after Ad-GFP or Ad-Cre infection. Boxplots represent the quantification from at least 750 telomeric fibers from a representative experiment (^∗^p < 0.05; ^∗∗∗∗^p < 0.0001; two-way ANOVA). (D–G) Representative images (D and F) and quantification of the frequency of interaction (E and G) between PCNA and TRF1 as determined by *in situ* PLA assay in cells treated with NU1025 (D and E) or transfected with the indicated siRNAs (F and G). Data represent quantification from at least 150 nuclei from a representative experiment (^∗∗∗∗^p < 0.0001; two-way ANOVA). Dashed lines indicate nucleus (as determined by DAPI in blue). (H) Quantification of the frequency of interaction between TERT and RAD51 as determined by *in situ* PLA assay in cells treated with NU1025. Data represent quantification from at least 150 nuclei from a representative experiment (^∗∗∗∗^p < 0.0001; two-way ANOVA). See also Figure S7.

To determine whether telomerase binding to reversed replication forks inhibits telomere replication in *Rtel1*-null cells, we employed SMARD (single-molecule analysis of replicated DNA) (Drosopoulos et al., 2015, Norio and Schildkraut, 2001, Sfeir et al., 2009) (Figure 7A). Quantification of CldU positive telomeres revealed a significant reduction in active replication forks in *Rtel1*^*−/−*^ *Terc*^*+/+*^ cells, when compared to control cells (Figures 7B and 7C). Deleting telomerase from RTEL1-deficient cells restored active telomere replication to levels comparable to wild-type controls. Together with our previous observations (Figures 3C and 3D), these data reveal that telomerase inhibits replication specifically at telomeres in the absence of RTEL1.

**Figure S7:**
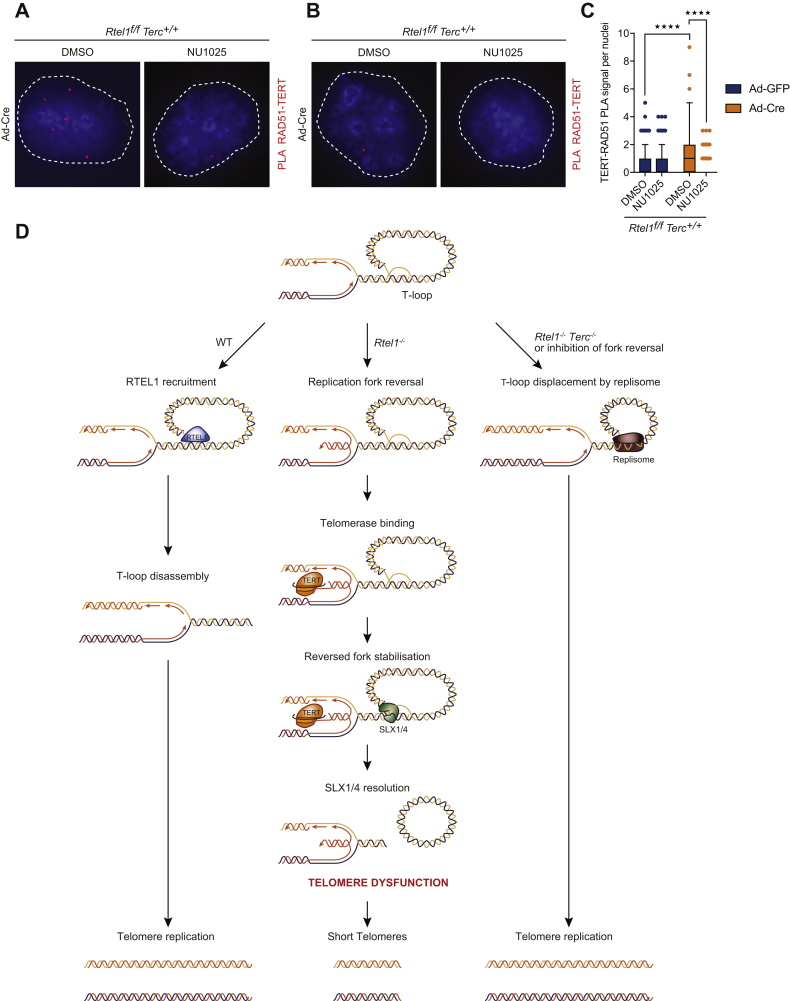
Telomerase Binding to Reversed Replication Forks Inhibits Replication at Telomeres, Related to Figure 7 (A) Representative images (from Figure 7H) of the interaction between TERT and RAD51 (using ab63801, Abcam) determined by *in situ* PLA assay in the different indicated treatments. Dashed lines indicate nucleus (as determined by DAPI in blue). (B and C) Representative images and quantification of the interaction between TERT and RAD51 (using PC130, Millipore) determined by *in situ* PLA assay in the different indicated treatments. Data represents quantification from at least 150 nuclei from a representative experiment (^∗∗∗∗^p < 0.0001; two-way ANOVA). Dashed lines indicate nucleus (as determined by DAPI in blue). (D) Schematic model of the impact of fork reversal and telomerase at telomeres in cells of the indicated genotypes. In WT cells, RTEL1 dismantles t-loops in S-phase to allow replication to occur unimpeded through the telomere. In Rtel1−/− cells, persistent T-loops hinder the replisome and trigger replication fork reversal within the telomere. The resulting single ended DSB of the reversed fork is bound by telomerase, which stabilize the reversed fork and blocks fork restart. In an attempt to remove the impasse to the replisome, SLX1/4 is recruited to the telomere where it cleaves off the t-loop resulting in the telomere dysfunction observed in Rtel1−/− cells. In cells lacking telomerase or inhibited for fork reversal, the replisome proceeds through the telomere and displaces the t-loop in its wake to complete telomere replication.

Together with the reduction in telomere replication in telomerase-proficient *Rtel1*^*−/−*^ cells, we predicted a corresponding increase in the frequency of stalled/reversed forks within telomeres. To test this possibility, we employed PLA to examine dysfunctional telomeres for the presence of PCNA, a key replication factor implicated in fork reversal (Vujanovic et al., 2017). A significant increase in PLA signal for TRF1-PCNA was evident at telomeres in the absence of RTEL1 (Figures 7D and 7E). The enhanced TRF1-PCNA PLA signal is dependent on reversed replication forks, as the PLA signal was largely eliminated by blocking fork reversal with PARP inhibitor (Figures 7D and 7E) or following downregulation of UBC13 or ZRANB3 (Figures 7F and 7G). Finally, PLA for TERT and RAD51, an essential component of the fork reversal machinery (Zellweger et al., 2015), revealed an enhanced association at RTEL1-deficient telomeres, which was dependent on fork reversal (Figures 7H, S7A, S7B, and S7C). Collectively, these results reinforce our conclusion that telomerase binds to reversed replication forks and hinders replication at telomeres in cells that lack RTEL1.

## Discussion

Telomerase solves the end replication problem by extending telomere repeats and is therefore essential for stem cell renewal and tissue homeostasis. However, telomerase is a “double-edged sword” as its re-expression in ∼90% of all human cancers is sufficient to drive transformation and provide unlimited proliferative capacity (Damle et al., 2004, Hultdin et al., 2003). Here, we make the unexpected discovery that telomerase is also the driver of telomere catastrophe in *Rtel1*-deficient cells. We establish that this pathological effect of telomerase results from its aberrant binding and stabilization of reversed replication forks within the telomere, which inhibits telomere replication. Once bound by telomerase, the only option to resolve the stalled replication fork is to recruit SLX1/4 to nucleolytically excise the offending DNA secondary structure, which results in dramatic consequences for the telomere.

Our conclusion that telomerase binds inappropriately to reversed replication forks that form within telomeres in *Rtel1*-null cells is supported by PLA and RNAscope experiments, which revealed that telomerase binds aberrantly to telomeres in these cells. PLA for TERT-RAD51 also placed telomerase in close proximity to reversed replication forks in the absence of RTEL1. Furthermore, blocking fork reversal by either PARP1 inhibition or knockdown of UBC13 or ZRANB3 abolished aberrant telomerase binding to telomeres in *Rtel1*-null cells. In turn, this prevented the accumulation of SLX1/4 at telomeres, which catalyzes t-loop excision, overcame the toxic effect of telomerase in *Rtel1*^*−/−*^ cells, and suppressed telomere dysfunction. We propose that blocking fork reversal prevents the fork from slowing and allows the replisome to replicate unimpeded through the telomere displacing the t-loop in its wake (Figure S7D).

The importance of fork reversal for this phenomenon is also supported by our analysis of fork restart activities in the context of *Rtel1* and telomerase deficiency. Depleting PARG or RECQ1, which inhibit fork restart (Berti et al., 2013), mimics the pathological effect of telomerase and is sufficient to induce telomere dysfunction in *Rtel1*^*−/−*^*Tert*^*−/−*^ cells (Figure S7D). Since loss of PARG or RECQ1 results in persistent/stabilized reversed forks (Berti et al., 2013), we hypothesized that binding of telomerase to the reversed fork would prevent efficient replication restart, possibly by outcompeting fork restart activities. In agreement with this possibility, analysis of replication dynamics at telomeres by SMARD demonstrated that telomerase inhibits active telomere replication in cells lacking RTEL1, whereas removing telomerase mitigates this effect. These results establish that telomerase binding to reversed replication forks is inhibitory for replication fork restart within telomeres.

It is important to consider that failure to unwind the t-loop in cells lacking RTEL1 could impact on telomerase function in two distinct ways: (1) it would restrict telomerase access to its normal substrate—the 3′ single-stranded G-overhang, and (2) by inducing fork reversal, a telomeric 3′ ssDNA overhang would be generated on the regressed arm of the reversed fork, which would mimic the normal substrate of telomerase but in the wrong location within the telomere. Hence, the inability to access its normal substrate together with the non-productive engagement of telomerase with the regressed DSB arm of the reversed fork would collectively hamper the ability of telomerase to solve the end replication problem. Such a scenario provides a likely explanation as to why extension of telomeres by telomerase is compromised in *mRtel1*-deficient embryonic stem cells (ESCs) (Uringa et al., 2012).

Biochemical studies have shown that telomerase also binds with high affinity to telomeric G4-DNA structures (Moye et al., 2015), which are unwound by RTEL1 *in vitro* (Vannier et al., 2013). Telomerase binding of these DNA secondary structures at a reversed fork could prevent their unwinding by RTEL1 and hinder fork restart leading to fragile telomeres. Stalling of the replisome at these telomeric DNA structures could be resolved by polymerase re-priming downstream of the obstacle/lesion (Yeeles and Marians, 2013), which would allow replication to continue to the end of the telomere. However, even a single fork stalling and re-priming event at a telomeric G4-DNA structure would leave a short region of un-replicated telomeric DNA, which could explain why telomere fragility manifests as smeared and/or multiple spatially distinct telomere FISH signals, consistent with gaps/unreplicated regions within the telomere. In addition, this could also explain why fragile telomeres are associated with anaphase bridges and mitotic catastrophe, which are hallmarks of common fragile sites and incomplete replication (reviewed in Gelot et al., 2015).

Our previous work established that the SLX1/4 nuclease is responsible for catastrophic processing of telomeres in the absence of RTEL1. We proposed at the time that nucleolytic processing of the t-loop by SLX1/4 results in telomere shortening/loss and length heterogeneity (Vannier et al., 2012). Our current findings raise an alternative scenario; it is the 4-way junction at the reversed fork that is processed by SLX1/4 rather than the 3-way junction at the t-loop. While the former is a possibility, several lines of evidence suggest that it is persistent t-loops rather than reversed forks that are processed by SLX1/4. First, biochemical evidence has shown that SLX1/4 preferentially cleaves a 3-way (e.g., t-loop) rather than a 4-way junction (e.g., reversed fork) (Wyatt et al., 2013). Second, replication fork collapse is generally attributed to the action of MUS81 not SLX1/4 (Wyatt et al., 2017). In this regard, we previously demonstrated that telomere dysfunction still occurs in *Rtel1*^*−/−*^*Mus81*^*−/−*^ MEFs, which lends further support to SLX4-dependent t-loop processing being the driver of telomere catastrophe as opposed to MUS81-dependent collapse of the reversed fork (Vannier et al., 2012).

Finally, our findings have implications for HHS patients harboring *Rtel1* mutations (Ballew et al., 2013a, Ballew et al., 2013b, Le Guen et al., 2013, Walne et al., 2013). HHS is a multi-systemic disorder associated with inter-uterine growth retardation, microcephaly, developmental delay, immunodeficiency, aplastic anemia, oral leukoplakia, nail dystrophy, and skin pigmentation anomalies, which reflect in part a stem-cell attrition problem in highly proliferative tissues. In light of our data, stem cells will be particularly susceptible to loss of RTEL1 as they possess high telomerase levels relative to differentiated tissues. Currently, the only available treatment for HHS patients is bone marrow transplantation, which mitigates bone marrow failure and immunodeficiency (Vogiatzi et al., 2013). Since the toxic effects of telomerase can be rescued by blocking fork reversal, our results raise the possibility that progression of HHS could be attenuated with PARP inhibitors, which are currently in clinical use for the treatment of homologous recombination-deficient breast and ovarian cancers.

## STAR★Methods

### Key Resources Table

**Table:** 

REAGENT or RESOURCE	SOURCE	IDENTIFIER
**Antibodies**		

Alexa Fluor 350-conjugated NeutrAvidin antibody	Thermo Fisher	Cat#A11236
Biotinylated anti-avidin antibody	Vector Laboratories	Cat#BA-0300; RRID: AB_2336108
Chicken Anti-Goat IgG (H+L) Antibody, Alexa Fluor594 Conjugated	Thermo Fisher	Cat#A-21468; RRID: AB_141859
Donkey Anti-Rabbit IgG (H+L) Antibody, Alexa Fluor488 Conjugated	Thermo Fisher	Cat#A-21206; RRID: AB_141708
Goat Anti-Mouse IgG (H+L) Antibody, Alexa Fluor555 Conjugated	Thermo Fisher	Cat#A-21422; RRID: AB_141822
Goat Anti-Rat IgG (H+L) Antibody, Alexa Fluor594 Conjugated	Thermo Fisher	Cat#A-11007; RRID: AB_141374
Mouse Anti-BrdU Monoclonal Antibody, FITC Conjugated, Clone B44	BD Biosciences	Cat#347583; RRID: AB_400327
Mouse Monoclonal anti-PAR	Enzo Life Sciences	Cat#ALX-804-220-R100; RRID: AB_2052275
Mouse Monoclonal anti-PARP1	Santa Cruz Biotechnology	Cat# sc-53643; RRID: AB_785086
Mouse Monoclonal anti-PCNA	Santa Cruz Biotechnology	Cat# sc-56; RRID: AB_628110
Mouse Monoclonal anti-TERT	Novus Biologicals	Cat#NB100-317; RRID: AB_2201588
Mouse Monoclonal anti-Tubulin	Sigma-Aldrich	Cat#T6074; RRID: AB_477582
Peroxidase-conjugated Goat anti-Mouse IgG (H+L)	Thermo Fisher Scientific	Cat#G-21040; RRID: AB_2536527
Peroxidase-conjugated Goat anti-Rabbit IgG (H+L)	Thermo Fisher Scientific	Cat#G-21234; RRID: AB_2536530
Rabbit Anti-Mouse IgG (H+L) Antibody, Alexa Fluor488 Conjugated	Thermo Fisher	Cat#A-11059; RRID: AB_142495
Rabbit monoclonal anti-SLX4	Santa Cruz Biotechnology	Cat#sc-135225; RRID: AB_10647085
Rabbit monoclonal anti-TPP1	Abcam	Cat#ab195234
Rabbit monoclonal anti-TRF2	Cell Signaling Technology	Cat#13136
Rabbit polyclonal anti-53BP1	Novus Biologicals	Cat#NB100-304; RRID: AB_10003037
Rabbit polyclonal anti-PARP	Cell Signaling Technology	Cat#9542; RRID: AB_2160739
Rabbit polyclonal anti-RAD51	Abcam	Cat#ab63801; RRID: AB_1142428
Rabbit polyclonal anti-RAD51	Millipore	Cat#PC130; RRID: AB_2238184
Rabbit polyclonal anti-RTEL1	Novus Biologicals	Cat#NBP2-22360
Rabbit polyclonal anti-TERT	Abcam	Cat#ab191523
Rabbit polyclonal anti-TRF1	Abcam	Cat#ab1423; RRID: AB_301006
Rabbit polyclonal anti-UBC13	Cell Signaling Technology	Cat#4919; RRID: AB_2211168
Rabbit polyclonal anti-Zranb3	Abclonal	Cat#A9555
Rat polyclonal anti-BrdU	Santa Cruz Biotechnology	Cat#sc-56258; RRID: AB_781696

**Chemicals, Peptides, and Recombinant Proteins**		

Adenovirus Ad-Cre-GFP	Vector Biolabs	Cat#1700
Adenovirus Ad-GFP	Vector Biolabs	Cat#1060
BIBR1532	Santa Cruz Biotechnology	Cat#sc-203843
Biotin-00-(TTAGGG)4 PNA probe	Discovery Peptides	Cat#F2006
CldU	Sigma-Aldrich	Cat#C6891
EDTA-free Complete protease inhibitor cocktail	Roche	Cat#COEDTAF-RO
FITC-TelC 5′-(CCCTAA)3-3′ PNA probe	PNA Bio-synthesis	Cat#F1009
GRN163L	Dr. Jerry Shay	N/A
IdU	Sigma-Aldrich	Cat#I7125
Low melting agarose	Sigma-Aldrich	Cat#A9414
NU1025	Sigma-Aldrich	Cat#N7287
Olaparib	Selleckchem	Cat#S1060
Phi29 DNA	Thermo Fisher	Cat#EP0091
PhosSTOP phosphatase inhibitor cocktail	Roche	Cat#PHOSS-RO
TAMRA-TelG 5′-(TTAGGG)3-3′ PNA probe	PNA Bio-synthesis	Cat#F1006

**Critical Commercial Assays**		

Duolink *In Situ* Red Starter Kit Mouse/Rabbit	Sigma-Aldrich	Cat#DUO92101
FiberPrep (DNA Extraction Kit)	Genomic Vision	Cat#EXTR-001
Lipofectamine RNAiMAX	Thermo Fisher	Cat#13778150
ProLong Gold antifade with DAPI	Thermo Fisher	Cat#P36931
QIAprep Spin Miniprep Kit	QIAGEN	Cat#27106
QuikChange Lightning Site-Directed Mutagenesis kit	Agilent Genomics	Cat#210518
RevertAid First Strand cDNA Synthesis Kit	Thermo Fisher	Cat#K1622
RNAscope	ACD	Cat#322350
RNeasy Mini Kit	QIAGEN	Cat#74106
SYBR Green PCR Master Mix	Thermo Fisher	Cat#4309155
TeloTAGGG Telomerase PCR ELISAPLUS	Sigma-Aldrich	Cat#000000012013789001
TeloTAGGG Telomere Length Assay kit	Sigma-Aldrich	Cat#000000012209136001

**Experimental Models: Mouse Strains**		

*Rtel1*^*f/f*^	Wu et al., 2007	N/A
*Terc*^*+/−*^	Lee et al., 2001	N/A

**Experimental Models: Cell Lines**		

Mouse Adult Fibroblasts *Rtel1*^*f/f*^*Terc*^*+/+*^ or *Terc*^*−/−*^	This study	N/A
Mouse Embryonic Fibroblasts *Rtel1*^*f/f*^	Vannier et al., 2012	N/A
Mouse Embryonic Fibroblasts *Trf2*^*F/-*^	Gift of Titia de Lange. Celli and de Lange, 2005	N/A

**Oligonucleotides**		

Individual: ON-TARGETplus Parg siRNA	Dharmacon	Cat#J-044091-09
Targeted Region:ORF		
Individual: ON-TARGETplus PARG siRNA	Dharmacon	Cat#J-044091-10
Individual: ON-TARGETplus Recql siRNA	Dharmacon	Cat#J-044778-09
Targeted Region:ORF		
Individual: ON-TARGETplus Recql siRNA	Dharmacon	Cat#J-044778-10
Targeted Region:ORF		
Individual: ON-TARGETplus Ube2n siRNA	Dharmacon	Cat#J-064604-09
Targeted Region:3′UTR		
Individual: ON-TARGETplus Ube2n siRNA	Dharmacon	Cat#J-064604-10
Targeted Region:3′UTR		
Individual: ON-TARGETplus Zranb3 siRNA	Dharmacon	Cat#J-044003-05
Targeted Region:Non-Coding,ORF		
Individual: ON-TARGETplus Zranb3 siRNA	Dharmacon	Cat#J-044003-06
Targeted Region:Non-Coding,ORF		
ON-TARGETplus Non-targeting Control Pool	Dharmacon	Cat#D-001810-10
SMARTpool: ON-TARGETplus Dkc1 siRNA	Dharmacon	Cat#L-059410
SMARTpool: ON-TARGETplus Parg siRNA	Dharmacon	Cat#L-044091-01
SMARTpool: ON-TARGETplus Parp1 siRNA	Dharmacon	Cat# L-040023-00
SMARTpool: ON-TARGETplus Recql siRNA	Dharmacon	Cat#L-044778-01
SMARTpool: ON-TARGETplus Tpp1 siRNA	Dharmacon	Cat#L-057987-01
SMARTpool: ON-TARGETplus Ube2n siRNA	Dharmacon	Cat#L-064604-01
SMARTpool: ON-TARGETplus Wrap53 siRNA	Dharmacon	Cat#L-051908-01
SMARTpool: ON-TARGETplus Zranb3 siRNA	Dharmacon	Cat#L-044003-00

**Recombinant DNA**		

LentiCRISPRv2	Dr. Feng Zhang lab. Sanjana et al., 2014	Cat#52961
pBABE-Hygro	Dr. Hartmut Land’s lab. Morgenstern and Land, 1990	Cat#1765
pLVX-TetOne	Takara	Cat#631847

**Software and Algorithms**		

Adobe Photoshop CC	Adobe	http://www.adobe.com/es/products/photoshop.html
GraphPad Prism 7	GraphPad	https://www.graphpad.com/
ImageJ	NIH	https://imagej.nih.gov/ij/
Volocity 6.3	PerkinElmer	http://cellularimaging.perkinelmer.com/downloads/detail.php?id=14

### Contact for Reagent and Resource Sharing

Further information and requests for reagents should be directed to and will be fulfilled by the Lead Contact, Simon J. Boulton (simon.boulton@crick.ac.uk).

### Experimental Model and Subject Details

#### Isolation of MAFs and Cell Culture Procedures

Source of cell lines used in the study is reported in the reagent and resource table. *Rtel1*^f/f^ mice (described in (Wu et al., 2007)) were crossed with early generation *Terc*^+/−^ mice (described in (Lee et al., 2001)). All mice were housed and maintained according to the Home Office guidance outlined in the Animal (Scientific Procedures) Act 1986. Mouse adult ear fibroblasts (MAFs) cell lines were derived from male aged matched *Rtel1*^*f/f*^*Terc*^*+/+*^ and *Rtel1*^*f/f*^*Terc*^*−/−*^ sibling mice. SV40-LT-immortalized as well as primary *Rtel1*^*f/f*^*Terc*^*+/+*^ and *Rtel1*^*f/f*^*Terc*^*−/−*^ MAFs, SV40-LT-immortalized *Rtel1*^*f/f*^ (Vannier et al., 2012) and *Trf2*^*f/-*^ (Celli and de Lange, 2005) a gift from Titia de Lange, The Rockefeller University) MEFs were cultured in Dulbecco’s modified Eagle’s medium (DMEM) supplemented with 15% fetal bovine serum (Invitrogen), L-glutamine, and penicillin-streptomycin. Deletion of floxed alleles in *Rtel1*^*f/f*^ and *Trf2*^*f/-*^ cells was carried out with either Ad-GFP or Ad-GFP-Cre adenovirus (Vector Biolabs). Cells were genotyped by PCR at 96 hr post-infection to confirm gene deletion. Olaparib (Selleckchem) and NU1025 (Sigma) were used at 5μM and 10μM, respectively, for 48 hours prior to cell collection. GRN163L (a gift from Jerry Shay, UT Southwestern) and BIBR1532 (Santa Cruz) were used at 2μM and 10μM, respectively, for 48 hours prior to cell collection.

### Method Details

#### Expression vectors

The mouse *Terc* cDNA was generated by PCR and subcloned into pLVX-TetOne lentiviral expression vector or into pBABE-Hygro retroviral expression vector. The mouse Tert cDNA was generated by PCR and subcloned into pBABE-Hygro retroviral expression vector. TERT mutants (T557M, K560N, K78E, and R132E) were then generated using the QuikChange Lightning Site-Directed Mutagenesis kit (Agilent Technologies) according to the manufacturer’s instructions. The generated mutants were verified by sequencing to screen against spurious secondary mutations.

#### *In Situ* Proximity Ligation Assay

Cells were plated on coverslips at density 5 × 10^4^ in 24-well plates and left in culture conditions overnight. The next day cells were pre-extracted in CSK buffer (10 mM PIPES [pH 6.8], 100 mM NaCl, 300 mM sucrose, 3 mM magnesium chloride, 1 mM EGTA, and 0.5% Triton X-100) fixed with 5% formaldehyde (Thermo Scientific) for 10 min, permeabilized with PBS containing 0.5% (v/v) NP-40 for 5 min, and blocked for 30 min with goat serum (5%) in PBS. PLA was performed following the manufacturer’s instructions using the Duolink anti-Mouse MINUS and anti-Rabbit PLUS *In Situ* PLA probes and the Duolink *In Situ* Detection Reagents Red (Olink Bioscience). Images were acquired with a Zeiss Axio Imager M1 microscope equipped with an ORCA-ER camera (Hamamatsu) and analyzed using Volocity 6.3 software (Improvision).

#### Cell lysis and western blotting

Cells were rinsed twice with PBS, transferred to ice-cold NET lysis buffer (50mM Tris (pH 7.2) 150 mM NaCl, 0.5% NP-40, 1x EDTA-free Complete protease inhibitor cocktail (Roche), 1x PhosSTOP phosphatase inhibitor cocktail (Roche) and lysed for 10 minutes on ice. The cell lysates were then briefly vortexed and passed through a 23G syringe five times. The soluble protein fractions were collected after centrifugation at 16000 x g for 10 minutes at 4°C. Protein lysates were analyzed by western blotting using standard SDS–polyacrylamide gel electrophoresis (SDS-PAGE) techniques. In brief, protein samples were boiled in Laemmli buffer, run in 4%–12% polyacrylamide gels, and transferred onto polyvinylidene difluoride membranes. The membranes were incubated overnight at 4°C with the appropriate primary antibodies. After being washed, the membranes were incubated with specific secondary horseradish peroxidase–linked antibodies from Dako and visualized using the enhanced chemiluminescence reagent from Amersham.

#### PNA FISH, Immunofluorescence-FISH, and RNAscope

Telomeric Peptide Nucleic Acid Fluorescence *In Situ* Hybrydysation (PNA FISH) was performed as described previously (Lansdorp et al., 1996). Briefly, cells were treated with 0.2 μg/ml of colcemid for 90 minutes to arrest cells in metaphase. Trypsinized cells were incubated in 75 mM KCL, fixed with methanol:acetic acid (3:1), and spread on glass slides. To preserve chromosome architecture the slides were rehydrated in PBS for 5 minutes, fixed in 4% formaldehyde for 5 minutes, treated with 1 mg/ml of pepsin for 10 minutes at 37°C, and fixed in 4% formaldehyde for 5 minutes. Next, slides were dehydrated in 70%, 85%, and 100% (v/v) ethanol for 15 minutes each and then air-dried. Metaphase chromosome spreads were hybridized with telomeric FITC-TelC 5′-(CCCTAA)3-3′ PNA probe or TAMRA-TelG 5′-(TTAGGG)3-3′ PNA probe (Bio-synthesis) and slides were mounted using ProLong Gold antifade with DAPI (Life Technologies). Chromosome images and telomere signals were captured using Zeiss Axio Imager M1 microscope equipped with an ORCA-ER camera (Hamamatsu) controlled by Volocity 6.3 software (Improvision). For IF-FISH, cells grown on #1.5 glass coverslips were fixed for 10 minutes in 2% (wt/vol) formaldehyde (Thermo Scientific). Cells were washed twice for 5 min in PBS, incubated for 30 min in blocking solution (1 mg/ml BSA, 3% goat serum, 0.1% Triton X-100, 1 mM EDTA in PBS), and then incubated overnight with primary antibody against 53BP1 or SLX4 and secondary antibody anti-mouse Alexa Fluor 488/555 secondary antibody (Molecular Probes) for 1 hr and 30 min, in blocking solution with 5 min washes in PBS in-between. After dehydration of the cells, FISH experiments were performed as described above. Slides were mounted with ProLong Gold antifade containing DAPI and images were acquired with an Olympus FLV1000 inverted microscope equipped with a 63X oil objective. Following acquisition, images were imported into ImageJ (NIH) and Adobe Photoshop CS5 for manual quantitation. RNAscope (Advanced Cell Diagnostics, Hayward, CA) was performed following the manufacturer instructions using a probe against mouse Terc RNA.

#### DNA combing

DNA combing was performed essentially as described in (Vannier et al., 2013). Briefly, *Rtel1*^*f/f*^*Terc*^*+/+*^ and *Rtel1*^*f/f*^*Terc*^*-/*^ MAFs were infected with control- or Cre-expressing adenovirus. Cells were pulse-labeled with IdU/CldU for 20 minutes, each pulse. DNA fibers were extracted in agarose plugs and stretched on silanized coverslips with the molecular combing system (Genomic Vision). CldU was detected with rat anti-BrdU antibody (BU1/75, AbCys), followed by goat anti-rat coupled to Alexa 594 (A11007, Molecular Probes) and finally by chicken anti-goat coupled to Alexa 594 (A21468, Molecular Probes). IdU was detected with Mouse anti-BrdU coupled to FITC antibody (BD44, Becton Dickinson), followed by rabbit anti-mouse coupled to Alexa 488 (A11059, Molecular Probes) and finally by donkey anti-rabbit coupled to Alexa 488 (A21206, Molecular Probes). DNA fibers were captured with a Zeiss Axio Imager M1 microscope equipped with an ORCA-ER camera (Hamamatsu) controlled by Volocity 6.3 software (Improvision).

#### siRNA and CRISPR

Cells were transfected with control siRNA or siRNA SMARTpool (Dharmacon) for the indicated gene 72 hours prior to cell collection using RNAiMAX (Thermo) following the manufacturer instructions. 24 hours after transfection media containing siRNA was substituted for fresh media. For the generation of CRISPR clones, guide RNAs for each gene were cloned into LentiCRISPRv2 vector. *Rtel1*^*f/f*^ MEFs were infected with the different gRNAs and selected with puromycin. Single cells were then seeded in 96-well plates until clones were large enough for genomic sequencing and TRAP assay.

#### Telomere Circle assay

Cells grown at a confluence between 70 to 80% were collected from two 10 cm dishes. To isolate genomic DNA, cells were then resuspended in TNE (10 mM Tris pH 7.4, 10 mM EDTA, 100 mM NaCl) and lysed in TNES (10 mM Tris pH 7.4, 100 mM NaCl, 10 mM EDTA, 1% SDS) in the presence of 100 μg/ml proteinase K. After overnight incubation with proteinase K at 37°C, and phenol/chloroform extraction, DNA was precipitated with isopropanol and resuspended in TE (10 mM Tris pH 7.5/1 mM EDTA). RNase A treatment, phenol/chloroform extractions and isopropanol precipitation followed. 3 μg of genomic DNA was digested with AluI/Hinf1 and resuspended in an annealing buffer (0.02 M Tris [pH 7.5], 0.02 M KCl, and 0.1 mM EDTA) with 1 μM Thio-TelC primer containing thiophosphate linkages between the three 3′ terminal nucleotides. The mix was denatured at 96°C for 5 min and cooled down to 25°C for 2 hr. DNA was ethanol precipitated and resuspended in 20 μL of the TCA reaction buffer (33 mM Tris-acetate [pH 7.9], 10 mM magnesium acetate, 66 mM potassium acetate, 0.1% Tween 20, 1 mM DTT, and 0.37 mM dNTPs). Primer extension was carried out with 7.5 U of ϕ29 DNA polymerase (Thermo Scientific) at 30°C for 12 hr. The ϕ29 DNA polymerase was inactivated by incubation at 65°C for 20 min. The extension products were separated by denaturing gel electrophoresis (0.8% agarose, 50 mM NaOH, and 1 mM EDTA [pH 8]) at 2 V/cm for 18 hr, transferred onto a nylon membrane, and Southern blotting membrane was hybridized with a γ[32P]-labeled Thio-TelC 5′-CCCTAACCCTAACCCTAAccc-3′ telomeric probe (small letters indicate two thiophosphate linkages between the three 3′ terminal nucleotides). Southern blot images were captured with a Storm 840 scanner. Telomere circle levels were quantified in Image J and were normalized to control reactions lacking Phi29 polymerase.

#### Telomere Length Analysis and Telomerase Repeated Amplification Protocol

Telomere length measurements were performed using the TeloTAGGG™ Telomere Length Assay kit from Roche and following manufacturer’s instructions. TRAP assay was performed using TeloTAGGG™ Telomerase PCR ELISAPLUS kit from Roche and following manufacturer’s instructions.

#### Quantitative RT-PCR

Cells were harvested and total RNA was isolated using TRIzol reagent following the manufacturer’s protocol. After digestion with RNase-free DNase I at 37°C for 30min, reverse transcription was carried out with 1 μg total RNA with random hexamer primers using RevertAid First Strand cDNA Synthesis Kit. Equal amounts of cDNA were mixed with iTaq SYBR Green Supermix and run on a Bio Rad CFX96 qPCR System. mRNA expression levels for the genes of interest were compared with B-Actin expression levels.

#### Single Molecule Analysis of Replicated DNA

The SMARD assay was performed essentially as described previously (Sfeir et al., 2009). Cells were labeled with 30 μM CldU for 4 hours, harvested and plugs containing 1x10^6^ cells were prepared in low melting agarose (Sigma). DNA plugs were processed for SMARD as described previously (Sfeir et al., 2009). DNA fibers were extracted from the plugs and combed onto silanized coverslips using the FiberPrep DNA extraction kit and the molecular combing system (Genomic Vision), according to the manufacturer’s instructions. Combed fibers were then denatured in alkali-denaturing buffer (0.1 N NaOH in 70% ethanol and 0.1% b-mercaptothanoland) for 12 minutes and fixed by adding 0.5% glutaraldehyde for 5 minutes. Telomeric DNA was identified by hybridizing with a Biotin-00-(TTAGGG)4 PNA probe and Alexa Fluor 350-conjugated NeutrAvidin antibody (Molecular Probes) followed by biotinylated anti-avidin antibody (Vector). Halogenated nucleotides were detected with a rat anti-BrdU monoclonal antibody (BU1/75, AbCys). Alexa Fluor 594-conjugated goat anti-rat (A11007, Molecular Probes) was used as a secondary antibody. Images were acquired using a Zeiss AxioImager M1, equipped with a Hamamatsu digital camera and the Volocity software (Perkin Elmer).

### Quantification and Statistical Analysis

Statistical parameters, including number of events quantified, standard deviation, and statistical significance are reported in the figures and in the figure legends. Statistical analysis has been performed using GraphPad Prism7 software (GraphPad) and statistical significance is determined by the value of p < 0.05 by Two-Way ANOVA test. Each experiment has been repeated at least twice.

## References

[bib1] Asai A., Oshima Y., Yamamoto Y., Uochi T.A., Kusaka H., Akinaga S., Yamashita Y., Pongracz K., Pruzan R., Wunder E. (2003). A novel telomerase template antagonist (GRN163) as a potential anticancer agent. Cancer Res..

[bib2] Ballew B.J., Joseph V., De S., Sarek G., Vannier J.B., Stracker T., Schrader K.A., Small T.N., O’Reilly R., Manschreck C. (2013). A recessive founder mutation in regulator of telomere elongation helicase 1, RTEL1, underlies severe immunodeficiency and features of Hoyeraal Hreidarsson syndrome. PLoS Genet..

[bib3] Ballew B.J., Yeager M., Jacobs K., Giri N., Boland J., Burdett L., Alter B.P., Savage S.A. (2013). Germline mutations of regulator of telomere elongation helicase 1, RTEL1, in Dyskeratosis congenita. Hum. Genet..

[bib4] Berti M., Ray Chaudhuri A., Thangavel S., Gomathinayagam S., Kenig S., Vujanovic M., Odreman F., Glatter T., Graziano S., Mendoza-Maldonado R. (2013). Human RECQ1 promotes restart of replication forks reversed by DNA topoisomerase I inhibition. Nat. Struct. Mol. Biol..

[bib5] Bétous R., Mason A.C., Rambo R.P., Bansbach C.E., Badu-Nkansah A., Sirbu B.M., Eichman B.F., Cortez D. (2012). SMARCAL1 catalyzes fork regression and Holliday junction migration to maintain genome stability during DNA replication. Genes Dev..

[bib6] Bétous R., Pillaire M.J., Pierini L., van der Laan S., Recolin B., Ohl-Séguy E., Guo C., Niimi N., Grúz P., Nohmi T. (2013). DNA polymerase κ-dependent DNA synthesis at stalled replication forks is important for CHK1 activation. EMBO J..

[bib7] Celli G.B., de Lange T. (2005). DNA processing is not required for ATM-mediated telomere damage response after TRF2 deletion. Nat. Cell Biol..

[bib8] Ciccia A., Nimonkar A.V., Hu Y., Hajdu I., Achar Y.J., Izhar L., Petit S.A., Adamson B., Yoon J.C., Kowalczykowski S.C. (2012). Polyubiquitinated PCNA recruits the ZRANB3 translocase to maintain genomic integrity after replication stress. Mol. Cell.

[bib9] Couch F.B., Bansbach C.E., Driscoll R., Luzwick J.W., Glick G.G., Bétous R., Carroll C.M., Jung S.Y., Qin J., Cimprich K.A., Cortez D. (2013). ATR phosphorylates SMARCAL1 to prevent replication fork collapse. Genes Dev..

[bib10] Damle R.N., Batliwalla F.M., Ghiotto F., Valetto A., Albesiano E., Sison C., Allen S.L., Kolitz J., Vinciguerra V.P., Kudalkar P. (2004). Telomere length and telomerase activity delineate distinctive replicative features of the B-CLL subgroups defined by immunoglobulin V gene mutations. Blood.

[bib11] de Lange T. (2005). Shelterin: The protein complex that shapes and safeguards human telomeres. Genes Dev..

[bib12] Denchi E.L., de Lange T. (2007). Protection of telomeres through independent control of ATM and ATR by TRF2 and POT1. Nature.

[bib13] Deng Z., Glousker G., Molczan A., Fox A.J., Lamm N., Dheekollu J., Weizman O.E., Schertzer M., Wang Z., Vladimirova O. (2013). Inherited mutations in the helicase RTEL1 cause telomere dysfunction and Hoyeraal-Hreidarsson syndrome. Proc. Natl. Acad. Sci. USA.

[bib14] Doksani Y., Wu J.Y., de Lange T., Zhuang X. (2013). Super-resolution fluorescence imaging of telomeres reveals TRF2-dependent T-loop formation. Cell.

[bib15] Drosopoulos W.C., Kosiyatrakul S.T., Schildkraut C.L. (2015). BLM helicase facilitates telomere replication during leading strand synthesis of telomeres. J. Cell Biol..

[bib16] Dungrawala H., Bhat K.P., Le Meur R., Chazin W.J., Ding X., Sharan S.K., Wessel S.R., Sathe A.A., Zhao R., Cortez D. (2017). RADX promotes genome stability and modulates chemosensitivity by regulating RAD51 at replication forks. Mol. Cell.

[bib17] Faure G., Revy P., Schertzer M., Londono-Vallejo A., Callebaut I. (2014). The C-terminal extension of human RTEL1, mutated in Hoyeraal-Hreidarsson syndrome, contains Harmonin-N-like domains. Proteins.

[bib18] Gelot C., Magdalou I., Lopez B.S. (2015). Replication stress in Mammalian cells and its consequences for mitosis. Genes (Basel).

[bib19] Gramatges M.M., Qi X., Sasa G.S., Chen J.J., Bertuch A.A. (2013). A homozygous telomerase T-motif variant resulting in markedly reduced repeat addition processivity in siblings with Hoyeraal Hreidarsson syndrome. Blood.

[bib20] Greider C.W., Blackburn E.H. (1985). Identification of a specific telomere terminal transferase activity in Tetrahymena extracts. Cell.

[bib21] Griffith J.D., Comeau L., Rosenfield S., Stansel R.M., Bianchi A., Moss H., de Lange T. (1999). Mammalian telomeres end in a large duplex loop. Cell.

[bib22] Harley C.B., Futcher A.B., Greider C.W. (1990). Telomeres shorten during ageing of human fibroblasts. Nature.

[bib23] Herbert B.S., Gellert G.C., Hochreiter A., Pongracz K., Wright W.E., Zielinska D., Chin A.C., Harley C.B., Shay J.W., Gryaznov S.M. (2005). Lipid modification of GRN163, an N3′-->P5′ thio-phosphoramidate oligonucleotide, enhances the potency of telomerase inhibition. Oncogene.

[bib24] Hultdin M., Rosenquist R., Thunberg U., Tobin G., Norrback K.F., Johnson A., Sundström C., Roos G. (2003). Association between telomere length and V(H) gene mutation status in chronic lymphocytic leukaemia: Clinical and biological implications. Br. J. Cancer.

[bib25] Karlseder J., Broccoli D., Dai Y., Hardy S., de Lange T. (1999). p53- and ATM-dependent apoptosis induced by telomeres lacking TRF2. Science.

[bib26] Lansdorp P.M., Verwoerd N.P., van de Rijke F.M., Dragowska V., Little M.T., Dirks R.W., Raap A.K., Tanke H.J. (1996). Heterogeneity in telomere length of human chromosomes. Hum. Mol. Genet..

[bib27] Le Guen T., Jullien L., Touzot F., Schertzer M., Gaillard L., Perderiset M., Carpentier W., Nitschke P., Picard C., Couillault G. (2013). Human RTEL1 deficiency causes Hoyeraal-Hreidarsson syndrome with short telomeres and genome instability. Hum. Mol. Genet..

[bib28] Lee K.H., Rudolph K.L., Ju Y.J., Greenberg R.A., Cannizzaro L., Chin L., Weiler S.R., DePinho R.A. (2001). Telomere dysfunction alters the chemotherapeutic profile of transformed cells. Proc. Natl. Acad. Sci. USA.

[bib29] Lemaçon D., Jackson J., Quinet A., Brickner J.R., Li S., Yazinski S., You Z., Ira G., Zou L., Mosammaparast N., Vindigni A. (2017). MRE11 and EXO1 nucleases degrade reversed forks and elicit MUS81-dependent fork rescue in BRCA2-deficient cells. Nat. Commun..

[bib30] Mijic S., Zellweger R., Chappidi N., Berti M., Jacobs K., Mutreja K., Ursich S., Ray Chaudhuri A., Nussenzweig A., Janscak P., Lopes M. (2017). Replication fork reversal triggers fork degradation in BRCA2-defective cells. Nat. Commun..

[bib31] Morgenstern J.P., Land H. (1990). Advanced mammalian gene transfer: High titre retroviral vectors with multiple drug selection markers and a complementary helper-free packaging cell line. Nucleic Acids Res..

[bib32] Moye A.L., Porter K.C., Cohen S.B., Phan T., Zyner K.G., Sasaki N., Lovrecz G.O., Beck J.L., Bryan T.M. (2015). Telomeric G-quadruplexes are a substrate and site of localization for human telomerase. Nat. Commun..

[bib33] Neelsen K.J., Lopes M. (2015). Replication fork reversal in eukaryotes: From dead end to dynamic response. Nat. Rev. Mol. Cell Biol..

[bib34] Neelsen K.J., Zanini I.M., Herrador R., Lopes M. (2013). Oncogenes induce genotoxic stress by mitotic processing of unusual replication intermediates. J. Cell Biol..

[bib35] Norio P., Schildkraut C.L. (2001). Visualization of DNA replication on individual Epstein-Barr virus episomes. Science.

[bib36] O’Sullivan R.J., Karlseder J. (2010). Telomeres: Protecting chromosomes against genome instability. Nat. Rev. Mol. Cell Biol..

[bib37] Palm W., de Lange T. (2008). How shelterin protects mammalian telomeres. Annu. Rev. Genet..

[bib38] Pascolo E., Wenz C., Lingner J., Hauel N., Priepke H., Kauffmann I., Garin-Chesa P., Rettig W.J., Damm K., Schnapp A. (2002). Mechanism of human telomerase inhibition by BIBR1532, a synthetic, non-nucleosidic drug candidate. J. Biol. Chem..

[bib39] Ray Chaudhuri A., Hashimoto Y., Herrador R., Neelsen K.J., Fachinetti D., Bermejo R., Cocito A., Costanzo V., Lopes M. (2012). Topoisomerase I poisoning results in PARP-mediated replication fork reversal. Nat. Struct. Mol. Biol..

[bib40] Ray Chaudhuri A., Ahuja A.K., Herrador R., Lopes M. (2015). Poly(ADP-ribosyl) glycohydrolase prevents the accumulation of unusual replication structures during unperturbed S phase. Mol. Cell. Biol..

[bib41] Sanjana N.E., Shalem O., Zhang F. (2014). Improved vectors and genome-wide libraries for CRISPR screening. Nat. Methods.

[bib42] Sarek G., Vannier J.B., Panier S., Petrini J.H., Boulton S.J. (2015). TRF2 recruits RTEL1 to telomeres in S phase to promote t-loop unwinding. Mol. Cell.

[bib43] Schlacher K., Christ N., Siaud N., Egashira A., Wu H., Jasin M. (2011). Double-strand break repair-independent role for BRCA2 in blocking stalled replication fork degradation by MRE11. Cell.

[bib44] Schmidt J.C., Dalby A.B., Cech T.R. (2014). Identification of human TERT elements necessary for telomerase recruitment to telomeres. eLife.

[bib45] Schmidt J.C., Zaug A.J., Cech T.R. (2016). Live cell imaging reveals the dynamics of telomerase recruitment to telomeres. Cell.

[bib46] Sfeir A., de Lange T. (2012). Removal of shelterin reveals the telomere end-protection problem. Science.

[bib47] Sfeir A., Kosiyatrakul S.T., Hockemeyer D., MacRae S.L., Karlseder J., Schildkraut C.L., de Lange T. (2009). Mammalian telomeres resemble fragile sites and require TRF1 for efficient replication. Cell.

[bib48] Shippen-Lentz D., Blackburn E.H. (1990). Functional evidence for an RNA template in telomerase. Science.

[bib49] Sogo J.M., Lopes M., Foiani M. (2002). Fork reversal and ssDNA accumulation at stalled replication forks owing to checkpoint defects. Science.

[bib50] Taglialatela A., Alvarez S., Leuzzi G., Sannino V., Ranjha L., Huang J.W., Madubata C., Anand R., Levy B., Rabadan R. (2017). Restoration of replication fork stability in BRCA1- and BRCA2-deficient cells by inactivation of SNF2-family fork remodelers. Mol. Cell.

[bib51] Takai K.K., Kibe T., Donigian J.R., Frescas D., de Lange T. (2011). Telomere protection by TPP1/POT1 requires tethering to TIN2. Mol. Cell.

[bib52] Thangavel S., Berti M., Levikova M., Pinto C., Gomathinayagam S., Vujanovic M., Zellweger R., Moore H., Lee E.H., Hendrickson E.A. (2015). DNA2 drives processing and restart of reversed replication forks in human cells. J. Cell Biol..

[bib53] Uringa E.J., Lisaingo K., Pickett H.A., Brind'Amour J., Rohde J.H., Zelensky A., Essers J., Lansdorp P.M. (2012). RTEL1 contributes to DNA replication and repair and telomere maintenance. Mol. Biol. Cell.

[bib54] van Steensel B., Smogorzewska A., de Lange T. (1998). TRF2 protects human telomeres from end-to-end fusions. Cell.

[bib55] Vannier J.B., Pavicic-Kaltenbrunner V., Petalcorin M.I., Ding H., Boulton S.J. (2012). RTEL1 dismantles T loops and counteracts telomeric G4-DNA to maintain telomere integrity. Cell.

[bib56] Vannier J.B., Sandhu S., Petalcorin M.I., Wu X., Nabi Z., Ding H., Boulton S.J. (2013). RTEL1 is a replisome-associated helicase that promotes telomere and genome-wide replication. Science.

[bib57] Vogiatzi P., Perdigones N., Mason P.J., Wilson D.B., Bessler M. (2013). A family with Hoyeraal-Hreidarsson syndrome and four variants in two genes of the telomerase core complex. Pediatr. Blood Cancer.

[bib58] Vujanovic M., Krietsch J., Raso M.C., Terraneo N., Zellweger R., Schmid J.A., Taglialatela A., Huang J.W., Holland C.L., Zwicky K. (2017). Replication fork slowing and reversal upon DNA damage require PCNA polyubiquitination and ZRANB3 DNA translocase activity. Mol. Cell.

[bib59] Walne A.J., Vulliamy T., Kirwan M., Plagnol V., Dokal I. (2013). Constitutional mutations in RTEL1 cause severe dyskeratosis congenita. Am. J. Hum. Genet..

[bib60] Weston R., Peeters H., Ahel D. (2012). ZRANB3 is a structure-specific ATP-dependent endonuclease involved in replication stress response. Genes Dev..

[bib61] Wu X., Sandhu S., Ding H. (2007). Establishment of conditional knockout alleles for the gene encoding the regulator of telomere length (RTEL). Genesis.

[bib62] Wyatt H.D., Sarbajna S., Matos J., West S.C. (2013). Coordinated actions of SLX1-SLX4 and MUS81-EME1 for Holliday junction resolution in human cells. Mol. Cell.

[bib63] Wyatt H.D., Laister R.C., Martin S.R., Arrowsmith C.H., West S.C. (2017). The SMX DNA Repair Tri-nuclease. Mol. Cell.

[bib64] Yeeles J.T., Marians K.J. (2013). Dynamics of leading-strand lesion skipping by the replisome. Mol. Cell.

[bib65] Zellweger R., Dalcher D., Mutreja K., Berti M., Schmid J.A., Herrador R., Vindigni A., Lopes M. (2015). Rad51-mediated replication fork reversal is a global response to genotoxic treatments in human cells. J. Cell Biol..

[bib66] Zhong F.L., Batista L.F., Freund A., Pech M.F., Venteicher A.S., Artandi S.E. (2012). TPP1 OB-fold domain controls telomere maintenance by recruiting telomerase to chromosome ends. Cell.

