# Supplementary material for: Stabilization of Reversed Replication Forks by Telomerase Drives Telomere Catastrophe
Source: Cell. 2018 Jan 25;172(3):439–453.e14. doi: 10.1016/j.cell.2017.11.047 (PMC5786504; doi:10.1016/j.cell.2017.11.047)
Supplement: Document S1. Table S1 [file mmc1.docx]

Table S1. Related to Figure 2.

| ***Terc*** | **WT** | ACCTGCAGCGGGCCACCGCGCGTTCCCGAGCCTCAAAAACAAACGTCAGCGCAGGAGCTC  CAGGTTCGCCGGGAGCTCCGCGGCGCCGGGCCGCCCAGTCCCGTACCCGCCTACAGGCCG  CGGCCGGCCTGGGGTCTTAGGACTCCGCTGCCGCCGCGAAGAGCTCGCCTCTGTCAGCCG  CGGGGCGCCGGGGGCTGGGGCCAGGCCGGGCGAGCGCCGCGAGGACAGGAATGGAACTGG  TCCCCGTGTTCGGTGTCTTACCTGAGCTGTGGGAAGTGCACCCGGAACTCGGTTCTCACA |  |
| --- | --- | --- | --- |
|  | Clone 1 | ACCTGCAGCGGGCC-----------------------------------------TCACA  ACCTGCAGCGGGCCACCGC-----------------------------------------  ---------TGGGGTCTTAGGACTCCGCTGCCGCCGCGAAGAGCTCGCCTCTGTCAGCCG | -281bp  -110bp |
|  | Clone 2 | ACCTGCAGCGGGCCACCGCGCGTTCCCGAGCCTCAAAAACAAACGTCAGCGC--------  ---------TGGGGTCTTAGGACTCCGCTGCCGCCGCGAAGAGCTCGCCTCTGTCAGCCG  ACCTGCAGCGGGCCACCGCGCGTTCCCGAGCCTCAAAAACAAACGTCAGCCTCAGGAGCT | -77bp  1S+1bp |
|  | Clone 3 | ACCTGCAGCGGGCCACCGCGCGTTCCCGAGCCTCAAAAACAAACGTCAGCGCAGGAGCT-  -----------------------------------CAGTCCCGTACCCGCCTACAGGCCG  ACCTGCAGCGGGCCACCGCGCGTTCCCGAGCCTCAAAAACAAACGTCAGCGCAGGAGCT-  ------------GGCTGGGGCCAGGCCGGGCGAGCGCCGCGAGGACAGGAATGGAACTGG | -36bp  -133bp |

| ***Tert*** | **WT** | GTGTCATCCCTGAAAGAGCTGGTGGCCAGGGTTGTGCAGAGACTCTGCGAGCGCAACGAG  AGAAACGTGCTGGCTTTTGGCTTTGAGCTGCTTAACGAGGCCAGAGGCGGGCCTCCCATG  GCCTTCACTAGTAGCGTGCGTAGCTACTTGCCCAACACTGTTATTGAGACCCTGCGTGTC  AGTGGTGCATGGATGCTACTGTTGAGCCGAGTGGGCGACGACCTGCTGGTCTACCTGCTG  GCACACTGTGCTCTTTATCTTCTGGTGCCCCCCAGCTGTGCCTACCAGGTGTGTGGGTCT  CCCCTGTACCAAATTTGTGCCACCACGGATATCTGGCCCTCTGTGTCCGCTAGTTACAGG  CCCACCCGACCCGTGGGCAGGAATTTCACTAACCTTAGGTTCTTACAACAGATCAAGAGC  AGTAGTCGCCAGGAAGCACCGAAACCCCTGGCCTTGCCATCTCGAGGTACAAAGAGGCAT  CTGAGTCTCACCAGTACAAGTGTGCCTTCAGCTAAGAAGGCCAGATGCTATCCTGTCCCG  AGAGTGGAGGAGGGACCCCACAGGCAGGTGCTACCAACCCCATCAGGCAAATCATGGGTG  CCAAGTCCTGCTCGGTCCCCCGAGGTGCCTACTGCAGAGAAAGATTTGTCTTCTAAAGGA  AAGGTGTCTGACCTGAGTCTCTCTGGGTCGGTGTGCTGTAAACACAAGCCCAGCTCCACA  TCTCTGCTGTCACCACCCCGCCAAAATGCCTTTCAGCTCAGGCCATTTATTGAGACCAGA  CATTTCCTTTACTCCAGGGGAGATGGCCAAGAGCGTCTAAACCCCTCATTCCTACTCAGC  AACCTCCAGCCTAACTTGACTGGGGCCAGGAGACTGGTGGAGATCATCTTTCTGGGCTCA |  |
| --- | --- | --- | --- |
|  | Clone 1 | AGTGGTGCATGGATGCTACTGT--------------------------------------  GCACACTGTGCTCTTTATCTTCTGGTGCCCCCCAGCTGTGCCTACCAGGTGTGTGGGTCT  AGTGGTGCATGGATGCTACTGTTGAGCCGAGTGGGCGACGACCTGCT-GTCTACCTGCTG | -38bp  -1bp |
|  | Clone 2 | GTGTCATCCCTGAAAGAGCTGGTGGCCAGGGTTGTGCAGAGACTCTGCGAGCGCAACGAG  AGAA-CGTGCTGGCTTTTGGCTTTGAGCTGCTTAACGAGGCCAGAGGCGGGCCTCCCATG  GTGTCATCCCTGAAAGAGCTGGTGGCCAGGGTTGTGCAGAGACTCTGCGAGCGCAACG--  ----------------------------------------ACCTGCTGGTCTACCTGCTG | -1bp  -162bp |
|  | Clone 3 | AGTGGTGCATGGATGCTACTGTTGAGCCGAGTGG-CGACGACCTGCTGGTCTACCTGCTG  AGTGGTGCATGGATGCTACTGTTGAGCC---------------TGCTGGTCTACCTGCTG | -1bp  -15bp |

Genomic sequencing analysis of *Terc* or *Tert* genes in the different clones generated showing base deletions (indicated with the number of bases lost), substituted bases (indicated with an S), or base insertions (indicated with the number of bases gained), as compared to the WT cells.
